# How does it affect service delivery under the National Health Insurance Scheme in Ghana? Health providers and insurance managers perspective on submission and reimbursement of claims

**DOI:** 10.1371/journal.pone.0247397

**Published:** 2021-03-02

**Authors:** Patricia Akweongo, Samuel Tamti Chatio, Richmond Owusu, Paola Salari, Fabrizio Tedisio, Moses Aikins

**Affiliations:** 1 University of Ghana, School of Public Health, Accra, Ghana; 2 Navrongo Health Research Centre, Navrongo, Ghana; 3 Department of Epidemiology and Public Health, Swiss Tropical and Public Health Institute (Swiss TPH), University of Basel, Basel, Switzerland; Institute of Mental Health, SINGAPORE

## Abstract

**Introduction:**

In 2003, the Government of Ghana launched the National Health Insurance Scheme (NHIS) to enable all Ghanaian residents to have access to health services at the point of care without financial difficulty. However, the system has faced a number of challenges relating to delays in submission and reimbursement of claims. This study assessed views of stakeholders on claims submission, processing and re-imbursement under the NHIS and how that affected health service delivery in Ghana.

**Methods:**

The study employed qualitative methods where in-depth interviews were conducted with stakeholders in three administrative regions in Ghana. Purposive sampling method was used to select health facilities and study participants for the interviews. QSR Nvivo 12 software was used to code the data into themes for thematic analysis.

**Results:**

The results point to key barriers such as lack of qualified staff to process claims, unclear vetting procedure and the failure of National Health Insurance Scheme officers to draw the attention of health facility staff to resolve discrepancies on time. Participants perceived that lack of clarity, inaccurate data and the use of non-professional staff for NHIS claims vetting prolonged reimbursement of claims. This affected operations of credentialed health facilities including the provision of health services. It is perceived that unavailability of funds led to re-use of disposable medical supplies in health service delivery in credentialed health facilities. Stakeholders suggested that submission of genuine claims by health providers and regular monitoring of health facilities reduces errors on claims reports and delays in reimbursement of claims.

**Conclusion:**

Long delays in claims reimbursement, perceived vetting discrepancies affect health service delivery. Thus, effective collaboration of all stakeholders is necessary in order to develop a long-term strategy to address the issue under the NHIS to improve health service delivery.

## Introduction

Health is an important factor in the socioeconomic development of every nation [1]. Following the World Health Organization’s recommendations for countries to adopt appropriate interventions to promote Universal Health Coverage (UHC) for their citizens, some Governments all over the world have adopted policies and measures to improve access to health care [2,3]. In Africa for instance, many countries have introduced health insurance schemes [3,4]. However, health insurance schemes are complex to govern and they require a strong administrative capacity and accountability mechanisms. Such accountability mechanisms if well instated and pursued allow the providers, payers and patients to offer and receive appropriate health care. Health care settings consist of various interactions among patients, providers, suppliers, regulators and other players. Since interactions among these groups define the health care experience, it reinforces the cooperation required for health services delivery [5].

The Government of Ghana launched the National Health Insurance Scheme (NHIS) in 2003 to replace user fees or what was popularly known as “cash and carry” system. The aim of the NHIS was to ensure that all Ghanaians are able to access the needed health care services [6]. The Scheme was established through an Act of Parliament (Act 650, revised to Act 852 in 2012) in 2003 to provide financial risk protection against the cost of basic health care for all Ghanaian residence. Under the NHIS, all credentialed health facilities have been mandated to provide health services to insured clients and then submit claims for re-imbursement [6]. Though, NHIS faces financial, managerial and many other challenges [7], the Scheme exempted certain categories of people such as the poor and vulnerable, children below 18 years, elderly above 70 years, pensioners of SSNIT (Social Security and National Insurance Trust) and people with mental health disorders [6,8]. The Scheme also included free maternal health care that allows all pregnant women to have free registration with the NHIS after which they would be entitled to free services throughout pregnancy, childbirth and three months postpartum period [9,10].

In Ghana, the key players in the health sector are the Ministry of Health, which formulates policy, the Ghana Health Service, which provides over 50% of health services, the Christian Health Association of Ghana that provides about 42% of healthcare [7]. Additionally, the pharmaceutical Society of Ghana plays an important role in health service delivery process [7]. Health service delivery has been decentralized to improve both access to health services and community involvement in planning and delivery of health care services. The Health care system in Ghana follows a three-tier arrangement: primary, secondary and tertiary levels. Similarly, there are three levels of management in the health sector in Ghana thus, national, regional and district levels [6,7].

While Ghana’s NHIS has been praised globally as an excellent example of promoting and improving access to health service delivery, the system has not been without challenges [11,12]. For instance, about 1% of all claims submitted by credentialed health facilities were settled between 31 and 60 days. Further, the proportion of claims settled beyond 90 days increased consistently from 26% in 2011 to 100% in 2014 respectively [13]. The lengthy delay of reimbursement of claims has been attributed to the inability of the NHIA to mobilize enough revenue to meet the increasing cost of medical claims [7,14,15]. As a result, a number of health care providers in private and public service threatened to withdraw health services provided to NHIS clients in 2014 [16,17]. This could possibly affect the aim and purpose of which the scheme was introduced. Research into understanding the submission, processing and reimbursement of claims as well as ways to facilitate claims reimbursement to credentialed health facilities could contribute significantly to improving efficiency and healthcare [18]. Therefore, this study explored stakeholders’ views on the issue of delays in submission and re-imbursement of claims and how it affects health service delivery in Ghana.

## Method

### Ethical considerations

The study protocol was reviewed and approved by the Ghana Health Service Ethics Review Committee (Approval number GHS-ERC: 012/03/18) before the commencement of the study. Written informed consent was obtained from all study participants before the interviews were conducted. The informed consent was obtained without coercion, undue influence or misrepresentation of potential benefits and risks associated with participation in the study. The interview moderators explained to all participants the purpose of the study, procedures and their right to take part or withdraw from the study if they so wish. Further, they were told about the benefits and risks to them as participants and efforts to ensure confidentiality of information collected. To further ensure that confidentiality of all participants enrolled in the study was protected, numeric codes were assigned to each participant and used in all study related documents instead of their names. In addition, the reports are reported in the aggregate and no one will be able to trace a particular response to the interviewee.

### Study design

The study employed a qualitative method where in-depth interviews (IDIs) were conducted with health care workers, health facility claims officers and National Health Insurance Authority officers in three regions of Ghana. Qualitative research is ‘descriptive’ of the process and the meanings gained through words help in capturing the feelings, experiences and perceptions of individuals on the issue under investigation [19]. This study adopted qualitative approach, which allowed study participants to share their views in more detail on the issues under investigation. The approach was deemed appropriate because the study aimed at gaining deeper understanding on how claims processing, submissions and reimbursement affected health service delivery under the Ghana National Health Insurance Scheme.

Inductive approach to research involves collecting data that is relevant to research topic of interest. Once substantial data have been collected, the researcher then looks for patterns in the data, working to develop a theory that could explain such patterns. Deductive approach on the other hand entails the researcher taking steps described earlier or studies what others have done, reads existing theories of whatever phenomenon he or she is studying, and then tests hypotheses that emerge from those theories. Based on these epistemological approaches, the study used inductive approach of data collection and analysis where patterns were developed from the data based on the issue under investigation [20,21].

Data collection was part of a wider study funded by the Swiss Research for Development (r4d), “Health systems governance for an inclusive and sustainable social health protection in Ghana and Tanzania” project. Two papers were developed using the same data set first to look at claims’ submission and re-imbursement of claims and how it affects health service delivery under the National Health Insurance Scheme in Ghana and the other paper looks at co-payment under the NHIS in Ghana. Therefore, the description of the method section of the two papers is similar.

### Study site

The study was conducted in three of the ten previous administrative regions in Ghana. Compared to other neighbouring countries, Ghana’s economy has seen some impressive growth although, not fairly distributed as geographic and socio-economic equalities are wide [22]. There is regional variation in poverty with the north experiencing higher poverty than the south [22,23].

Specifically, the study was carried out in Central, Ashanti and Northern regions of Ghana. Central region covers an area of 9,826 square kilometres with an estimated population of 2,201,863. Ashanti region on the other hand covers an area of 24,389 square kilometres with an estimated population of about 4,780,380 while Northern region covers an area of 70,384 square kilometres with a population of 2,479,461 [24]. In terms of health care facilities, Central region has a total of about 415, Ashanti region 1,353 and Northern region 365 public and private health facilities [25].

### Sampling technique/Study participants

Purposive sampling procedure allows the researchers to choose study participants who they think are appropriate for the study [26]. This is a non-probabilistic sampling procedure, which is widely used in qualitative research and is appropriate to identify and select information-rich cases or participants for a study [27]. Therefore, purposive sampling technique was used to select three regions, health facilities and study participants. Central region is located in the Southern belt while Ashanti and Northern regions are located in the Middle and Northern belts of Ghana respectively. These regions were selected to enable the study team solicit views from participants across the different geographical belts in the country. Health providers/managers (i.e medical superintendents, medical assistants, hospital administrators and accountants), claims officers working in the selected health facilities and National Health Insurance Authority (NHIA) officers in the three regions were also purposively selected and interviewed after informed consent was obtained. First of all, letters were written to the heads of health facilities selected for the study and NHIA explaining the rationale of the study and also asking for their permission to conduct interviews with these categories of staff. After permission was obtained, data collectors visited these facilities and NHIA offices and participants who were available and agreed to take part in the study were recruited.

These category of health providers and claims offices are directly involved in preparation and submission of claims while NHIA mangers/officers validate and reimburse claims to credentialed health facilities. Thus their views on delays in submission, processing and imbursement of claims were sought. The main themes explored included: knowledge on guidelines for processing and submission of claims, experiences in preparing claims, submission, processing and reimbursement as well as suggested ways to improve reimbursement of claims and provision of health care.

### Training and data collection procedures

Six university graduates with experience in conducting qualitative interviews were recruited and trained for data collection. The training lasted for two weeks, which covered areas such as the purpose and objectives of the study, qualitative interviewing techniques and the responsibilities of data collectors in the study. Role plays were done during the training that helped data collectors have clearer understanding of the interview guides. Pre-test was also conducted at the end of the training to evaluate the performance of data collectors and that also enabled the study team to finalize the interview guides before the actual data collection started.

In-depth interviews guides were designed and used to conduct the interviews with participants. Within each region, a tertiary hospital, regional/municipal hospital, district hospital, three health centre in-charges and two Community-based Health Planning and Services in-charges were interviewed. At the tertiary, regional, municipal and district levels, the Medical Director in-charge of the hospital, the Administrator, the Claims Officer and Accountant were each interviewed. National Health Insurance Authority (NHIA) officers working in these regions were also interviewed. Appointments were made with participants on suitable date, venue and time before the interviews were conducted. All interviews were conducted in the offices of participants. Each interview lasted for about 40 minutes. All the interviews were recorded with the consent of participants. The interviews were conducted in English from April 2018 to June 2018. In all, 66 in-depth interviews (22 in each region) were conducted.

### Data processing and analysis

The recorded interviews were transcribed verbatim by independent people with experience in transcribing qualitative interviews. Based on the objectives of the study and new themes that emerged from the data, a codebook was prepared to guide data coding and analysis. The recorded interviews were transcribed simultaneously with data collection. The study team read these transcripts and offered feedback to data collectors. This was to ensure that thematic saturation was monitored and that data was collected to cover all themes. The data was coded using QSR Nvivo 12 software for thematic framework analysis. In order to ensure a fair interpretation of the data, the transcripts were coded independently by two persons with extensive experience in qualitative research methodology including the use of QSR Nvivo to code and analyse qualitative data. This involved a careful review of each transcript to identify emerging themes from the data and also based on the objectives of the study. The two coders then met to compare their independently-identified themes. They resolved any divergence by re-reading the relevant sections of the transcripts together, and agreed on the best fit interpretation of the data. The research team then reviewed the coding systems and analysed the data thematically. The findings are presented and supported by relevant quotes from the data.

## Results

### Knowledge on guidelines for processing and submission of claims

There is general misunderstanding on the processes of submission of claims under the Ghana National Health insurance Scheme. From the results, study participants outlined different dates and submission procedures. While some participants were of the view that claims were manually processed, some of them reported that claims were processed electronically and sent directly to the NHIA (National Health Insurance Authority) office at the national level for reimbursement. Similar divergent views were observed with dates for claims submission such that while some participants submitted on monthly basis, others maintained that claims were submitted quarterly for reimbursement. Others held the view that the accredited health facilities had to submit claims reports to the NHIA district office through to the Regional office and finally to the national level.

*“What I know is that*, *claims are submitted within the second week of every month to the National Health Insurance Scheme office for reimbursement*.*”*

(IDI- Medical Superintendent-Central Region)

*“We are supposed to submit claims from 1*^*st*^ *to 15th of every month*. *We are doing it electronically so once we key in data*, *it goes to Accra directly (refers to NHIA office at the national level)*. *But those who are not using the electronic system must submit it within two weeks of every month*.*”*

(IDI- Health Service Administrator-Ashanti Region)

Some of the health workers said that generic names of drugs, signs and symptoms used to diagnose medical condition were the key things captured in the claims report.

*What happens is that*, *you have to add generic names of all drugs used and the signs and symptoms of illnesses treated*.*”*

(IDI- Medical Superintendent-Northern Region)

Few of the clinical health care workers reported that they did not have any knowledge regarding the guidelines for claims submission as illustrated in the quotes below:

*I*: *Do you know the guidelines for claims submission*?

*R*: *No please I don’t have an idea about such processes*.

(IDI- Midwife-Central Region)

*R*: *I do not know anything about claims guidelines because that is the work of the Pharmacist*.

(IDI- Midwife-Ashanti Region)

Some of the health facility claims officers and NHIA officers said that electronic software was used to submit claims quarterly to the NHIA claims processing centre (CPC) for re-imbursement. They explained that a cover letter (using a letter head with official stamp) and a summary sheet were supposed to be attached to the claims report before submission otherwise the claims report could be rejected.

*“When you finish with the claims*, *reports*, *you add a cover letter and send the claims through internet to the claims office*. *Also*, *you have to send the soft copy on a CD rom to the claims’ office*. *There is a format for the cover letter and if you don’t follow that format*, *they will reject your submissions*. *There is a summary sheet that should be attached to the cover letter*. *Also*, *if there is no stamp on your letter and if there is no letter head too*, *they will not accept it*.*”*

(IDI-Claims Officer-Central Region)

### Experiences in preparing claims, submission, processing and reimbursement

Study participants shared their experiences on the challenges in preparing claims, submission, processing, delays in re-imbursement of claims and how that affected health service delivery.

### Experiences in preparing and submission claims

The study sought to unravel the experiences health providers held in preparing and submission of claims to NHIA. Most of the health workers mentioned inadequate staff to process claims reports as the main problem they faced. They explained that health facilities relied mainly on National Service personnel (freshly graduated university students providing one-year service to the country) to process claims and it was a problem each time their service period ended. According to health providers, lack of qualified officers to prepare claims led to errors on claims reports submitted to NHIA for re-imbursement. Other challenges such as unclear vetting procedures by officers at the claims processing centre (CPC) and frequent change of claims forms were reported.

*I*: *What are the challenges you face in claims submission*?

*R*: *We have inadequate staff here*. *Most of our NHIS claim staff are national service personnel and casual workers*. *Every year they are supposed to leave and you need other new ones to train them again*

*I*: *How does it affect claim submission*?

*R*: *It distracts the process and it sometimes increases the level of mistakes because they are not qualified staff*.

(IDI- Head of Finance-Central Region)

*R*: *Yes*, *sometimes you take it (Referring to claims reports) to them (referring to NHIS office) and they tell you this particular disease does not go with this drug and this drug is been given overdose and they won’t pay*. *Quite apart from that we don’t even know the vetting procedures being used by NHIA on claims reports and that is also a problem for us*.

(IDI-Midwife-Northern Region)

The manual processing of claims was also mentioned by health workers in some of the credentialed health facilities as a challenge. They reported that a number of errors and duplications were made on claims reports due to this method of processing claims.

*“You know we see a lot of people and you have to prepare claims reports manually and it is so cumbersome*. *There are lots of human errors and duplication of figures…*.*”*

(IDI- Accountant-Ashanti Region)

Other challenges highlighted by some of the health providers included ineligible handwriting by prescribers and the use of wrong generic names of drugs. They explained that the hand writing of some prescribers was extremely difficult to read by staff in-charge of processing claims at the health facility level, which resulted in the errors made on claims reports.

*“Some of them don’t use the right generic names during the inputting phase and at times the diagnosis will be wrongly inputted*. *Also*, *the handwritings of the prescribers is a problem and it is sometimes difficult to read it*.*”*

(IDI- Director of Nursing-Ashanti Region)

Lack of qualified staff to process claims both at the health facility and CPC, ineligible and short handwriting by prescribers were reinforced by the NHIA staff and facility claims officers when interviewed. They said that some prescribers treating clients outside the NHIS medicine list also led to late re-imbursement of claims.

*“Most of them are casual workers and others use the National Service personnel to prepare claims reports and this creates lots of problems*. *Also*, *the vetting team at the NHIS CPC doesn’t have clinicians there*. *They only trained them to match 1 to 2 so if it is matched to 3*, *they complain it is miss-match since they are not clinicians and that leads to the deductions*. *Also*, *at the facility*, *the team working on claims submission makes mistakes due to the short hand writing by the clinicians*. *They write malaria as Mal or UTI and the new personnel at the CPC don’t know these things*. *The NHIS will not know the UTI as Urinary Tract infections*.*”*

(IDI- Claims Officer-Ashanti Region)

*“…some health providers also treat clients outside the medicine list*, *they go contrary to the medicine list and we have a clinical audit team that frequently goes around to point out some of these challenges at the health facilities*.*”*

(IDI- Manager-NHIS)

### Delays in reimbursement of claims and how it affects health service delivery

Most of the health providers reported that there were long delays in reimbursement of claims. They complained of lack of feedback as the reasons for these delays and in some instances, claims submitted were rejected without any explanation. Study participants lamented that it could take a year or two without a facility receiving any reimbursement of claims from the NHIA. The problem of claims reimbursement was reported by study participants in all the three regions.

I: When was the last time you received reimbursement from NHIA?

*R*: *Hmmm*, *it is a problem because they only managed to clear 2016 backlog*, *but we still have the whole of 2017 and 2018 claims to be paid*.

(IDI- Medical Superintendent-Central Region)

*R*: *Reimbursement is a serious problem for us because you will provide the services to NHIS clients and they will not pay you to enable you to continue providing the services to clients*. *I must say that it has been over ten months and no payment has been received from NHIA*.

(IDI- Medical Superintendent- Northern Region)

*R*: *So far*, *they have only paid for 2017 claims and we are still left with January to December*, *2018 claims to be paid*. *As we speak*, *I don’t know when they are going to make such payments*.

(IDI-Medical Director)

According to the health care providers, the delays in claims reimbursement had affected the operations of credentialed health facilities to the extent that they could not pay their suppliers to receive medical items such as medicines, logistics and other equipment to facilitate health service delivery. According to participants, the situation had made health providers to adopt the method of re-using disposal medical supplies for health service delivery in NHIS credentialed health facilities. The difficulty for health providers to pay casual workers engaged to support health service delivery in credentialed health facilities was also reported.

*“If the payment delays*, *we are not able to pay our suppliers*, *who also find it difficult to supply our drugs and this leads to shortage of drugs*. *It also delays payment of casual workers and a hungry man is an angry man*. *Now government does not engage laborers and we pay them from our IGF (Internal Generated Fund)*, *so if insurance delays then it’s difficult to pay them and also take care of other administrative costs and all these affect our ability to provide the health services to clients*.*”*

(IDI- Head of Finance-Central Region)

*“The inability of the NHIA to pay us really affects health care*. *In the past*, *after injecting someone*, *you throw both syringe and needle away but now we throw just the needle and re-use the syringe because there is no money to buy them*. *We don’t even have money to buy common gloves and use because health insurance has not reimbursed us*.*”*

(IDI- Pharmacist-Ashanti Region)

Although, the NHIA officers admitted there were delays to reimburse credentialed health facilities, they attributed it to the inability of the health providers to submit their claims on time and also the errors made in the claims report. They also attributed this to the volume of work at the claims processing centre coupled with lack of officers to vet claims submitted, further causing delays in reimbursement of claims.

*“You have to provide the services and once you provide the services*, *you bring the claims because we are not the ones to chase you for them*. *So*, *once it comes to us*, *we have to pay but most of the problems we are facing in the Northern region here have to do with the delay in submission claims*. *Secondly*, *improper processing of claims by the health facilities delays the process*.*”*

(IDI- Manager-NHIS)

*“Actually we know this affects the quality of health care…*.*But sometimes the challenges also come from the providers because they delay to submit their claims…*.*So*, *once you delay*, *then our claims processing center also delays*. *So partly*, *the problem is from the providers and not from the NHIA even though we know there are challenges with regard to staff to vet these claims before payments are made*.*”*

(IDI-Manager-NHIS)

The health facility claims officers also blamed health providers for lack of clarity and for submitting inaccurate data for claims reimbursement. They explained that some of the credentialed health facilities provide fake information to NHIA and that led to delays in reimbursement of such claims. As one of them put it:

*“There is delay alright from NHIS but the fault is from the providers*. *Most of them provide fake information to NHIS and that delays the payment*. *After submitting the claim*, *the policy is that it takes three months before payments are made*.*”*

(IDI- Claims Officer-Ashanti Region)

### Suggested ways to improve reimbursement of claims and provision of healthcare

#### Upward review of NHIS premium

Health providers suggested a review of the premium to allow NHIS members to pay realistic fees to sustain the scheme. They said this was the only way to help NHIA to raise the needed revenue to fund activities of the NHIS including reimbursement of claims. Health providers advised that the NHIS money should not be paid into the consolidated funds where Government could use it to fund other projects instead of the intended purpose of funding NHIS activities. According to them, this would help solve the unnecessary delays of reimbursement of claims.

*“NHIA should look at the premium and let people pay realistic amounts to enable them get the needed health services*. *The NHIS levy should be used for the intended purpose and it should not be added to the consolidated funds where it is used for other things*. *If the revenue collected for national health insurance are put in the NHIS fund and used correctly*, *they could be able to pay providers on time to improve health care*.*”*

(IDI- Medical Superintendent–Ashanti Region)

### Verification of claims and intensive supervision

Health care providers suggested that verification of claims should be done on facility by facility basis to facilitate rapid reimbursement of claims. They endorsed the need for frequent visits to credentialed health facilities by NHIS monitoring and evaluation team to help reduce errors made on claims reports. A Medical Superintendent put it as:

*“For the health insurance to work well*, *they need to assess the health seeking behaviours of clients*. *They should also verify claims facility by facility and not put all facilities together and lament that they are not doing well*. *They have a monitoring unit but they are not doing their work well*. *They will not visit the facilities to assess the folders and rather sit somewhere and say some facilities are thieves and for me*, *that is not the best*.*”*

(IDI- Medical superintendent-Ashanti Region)

Health facility claims staff also suggested the need for a software to enable NHIA track the use of health services by insured clients to ensure that claims submitted were genuine. They added that an electronic tracking system of services patients receive could help prevent situations where insured clients would move from one facility to another with the same illness for treatment, which they perceived increases the cost of medical care.

*“NHIS does not have a system to tract and check their clients and treatment behaviour*. *It is only when claims are submitted that NHIS knows a client has utilized services*. *We need one platform where if a client visits a facility and keys in*, *it shows that such services have been provided to the client*. *There is nothing like that in the system to track transactions*.*”*

(IDI- Claims Officer-Ashanti Region)

The NHIS managers reiterated the need for all stakeholders especially health providers to be transparent in providing the right services with corresponding claims submitted for re-imbursement to ensure the sustainability of the NHIS.

*“Actually*, *the sustainability of the scheme depends on all of us*. *It does not depend only on the health insurance management*, *it depends on all the major stakeholders especially health providers*. *They have a special role to play to ensure the sustainability of the scheme because they are providing the services and they have to ensure that the right thing is done by ensuring that the right services are provided and genuine claims are also submitted to NHIA*.*”*

(IDI- Manager-NHIS)

## Discussion

This study explored health providers/managers and insurance managers perspectives on claims submission, processing and reimbursement under the National Health Insurance Scheme of Ghana. The study assessed delays in claims reimbursement and how that affects health service delivery. The results point to some variations in claims processing and submission by health providers. Our data suggest that there is no uniformity in the procedure in use for processing and submission of claims. This suggests that enough education has not been given to health providers on the guidelines governing preparations and submission of claims for reimbursement.

The use of unqualified staff coupled with manual processing of claims has been described by study participants as responsible for the errors on claims reports. This practice is perceived to have contributed significantly to delays in reimbursement of claims according to health providers. Our results collaborate with earlier studies that demonstrated that administrative capacity, technical and human resource challenges contributed to delays in claims submission, vetting processes and reimbursement [7,28]. Other challenges such as unclear vetting procedure and the failure of Claims Processing Centre staff to draw the attention of health facilities to resolve errors on time were reported. This suggests that credentialed health facilities have not been educated on how to prepare claims based on the procedure being used to vet these claims. Earlier studies have reported high rates of non-reimbursement of claims due to errors in claims processing, lack of feedback regarding errors committed and unclear claims reporting procedures [7,16].

Our data suggest that the core challenge of the Ghana National Health Insurance Scheme is delay in reimbursement of claims. Our finding collaborates with current literature reporting that the main problem encountered by health providers in health service delivery was delays in reimbursement of claims [29]. Health providers in this study complained about how the long delays in claims reimbursement negatively affects their operations including the provision of health services to NHIS clients. The situation has affected their financial capacity to the extent that they could not purchase medical items to facilitate health service delivery. This lapse has made credentialed health facilities to adopt the method of re-using medical supplies in the health service delivery process with its attendant safety issues. It is revealed that infection is one of the greatest safety risks with issues of re-use of medical supplies [30].

Although, some of the credentialed health facility claims staff and NHIA officers reinforced the issue of delay of reimbursement of claims, they attributed it to the perceived inability of health providers to submit their claims on time and the errors made on claims reports. Further, the perceived health provider fraud in submission claims leads to suspicion of the validity of claims submitted, which delays vetting process and re-imbursement of claims. This is consistent with an earlier study in Ghana reporting that lack of transparency among healthcare providers led to distrust and delays in reimbursement of claims by health insurance authorities [31]. It has also been established that lack of transparency among providers of healthcare could lead to inefficiency and rising costs of health services [31,32]. This means that open relations with healthcare and insurance providers are based on healthcare providers’ clear communication, attitude and reliability of services as well as their ability to be transparent in claims reports submitted for reimbursement.

Evidence exists that about 22% of all claims submitted by health providers in Ghana were settled between 31 and 60 days in 2011 and none of the claims was settled within the same period in 2013 [13]. Delay in reimbursement of claims has been due to the inability of NHIA to mobilize enough revenue to meet the increasing cost of medical claims [14]. Other studies in Ghana also found that about 81% of credentialed health facilities’ revenue is generated through provision of services to NHIS clients [33,34]. Therefore, delays in claims settlement distorted the financial capacity and operations of health service providers thereby affecting health service delivery. In the long-run, this could negatively affect enrolment and growth of the Scheme. This could also lead to existing providers withdrawing the services. This would discourage other service providers from joining the pool of credentialed providers in the provision of health services to the increasing number of insured clients thus affecting equity and geographical access to health care services in Ghana [13,15].

Also, Government interference in the disbursement and payment of claims and broad exemptions are also contributory factors to the delay in reimbursement of claims under the Ghana National Health Insurance Scheme [15]. In the midst of the numerous challenges affecting health service delivery due to long delays in claims reimbursement by NHIA, study participants proposed various ways to improve financial capacity of NHIS to reduce delays of reimbursement of claims also sustain operations of the scheme. They opine that the NHIS money should be paid directly into the National Health Insurance Fund (NHIF) to prevent the delay in releasing money from the consolidated fund for the activities of the NHIS. Inadequate funding sources and insufficient premium have been described as major challenges of financing health care under NHIS in Ghana [35]. Study participants suggested that upward review of premium could help the NHIA to generate revenue to improve the financial capacity of the Scheme to reduce the delays in claims reimbursement and also help to sustain national health insurance scheme in Ghana.

## Strengths and limitations

The main strength of this research is the representation of health providers in credentialed health facilities and NHIA staff. This allowed us to obtain views from these stakeholders directly involved in health care delivery on issues that affect delays in submission, vetting, reimbursement of claims and health service delivery. The main limitation was that the study used only qualitative methods to assess views on claims and its processes among health providers and NHIA staff. This design did not allow us to measure the magnitude of the effect claims delays and processing on health care among these stakeholders in-charge of health service delivery in Ghana. Another limitation is that the study used a non-probability (purposive) sampling method to select participants in the three regions to share their experiences on processing and reimbursement of claims which may not necessarily represent views of the larger population. Thus, the findings of this study may not be generalized to the larger population in Ghana.

## Conclusion

Based on the interpretation of the data, the introduction of the NHIS has improved access to health care services and quality of life for most people who otherwise could not afford health care services thus promoting the goals of universal health coverage. Undue delays of reimbursement of claims, threaten the ability of the scheme to perform adequately. This development negatively affects the provision of health services to insured clients under the NHIS. As the health needs of people continue to increase, costs are expected to rise accordingly. Thus, effective collaboration of all stakeholders is necessary in order to develop a long-term strategy to reduce costs and long delays in claims reimbursement to credentialed health facilities and improve its health service delivery mandate.

## Supporting information

S1 File (DOCX)

S2 File (ZIP)

S3 File (DOCX)

S4 File (DOCX)
