# Correction: How does it affect service delivery under the National Health Insurance Scheme in Ghana? Health providers and insurance managers perspective on submission and reimbursement of claims

**DOI:** 10.1371/journal.pone.0253357

**Published:** 2021-06-10

**Authors:** Patricia Akweongo, Samuel Tamti Chatio, Richmond Owusu, Paola Salari, Fabrizio Tediosi, Moses Aikins

The fifth author’s name is spelled incorrectly. The correct name is: Fabrizio Tediosi. The correct citation is: Akweongo P, Chatio ST, Owusu R, Salari P, Tediosi F, Aikins M (2021) How does it affect service delivery under the National Health Insurance Scheme in Ghana? Health providers and insurance managers perspective on submission and reimbursement of claims. PLoS ONE 16(3): e0247397. https://doi.org/10.1371/journal.pone.0247397
