# Supplementary material for: How does it affect service delivery under the National Health Insurance Scheme in Ghana? Health providers and insurance managers perspective on submission and reimbursement of claims
Source: PLoS One. 2021 Mar 2;16(3):e0247397. doi: 10.1371/journal.pone.0247397 (PMC7924798; doi:10.1371/journal.pone.0247397)
Supplement: S2 File — (ZIP) [file pone.0247397.s002.zip › S1 File. Study aata/Health providers and Managers/Challenges.docx]

[<Internals\\Health care providers\\IDI-Facility In-charge>](42a21635-e6b2-4991-a5d6-3deeba398cc3) - § 1 reference coded [4.43% Coverage]

Reference 1 - 4.43% Coverage

I What has been some of the challenges with claim system?

R After we have submitted to the hospital, we don’t get any feedback from them on the amount we got from the claims submitted. So when we request they will tell us they will get the figure for us but they will not get back to us with the feedback and that is the major problems we face.

[<Internals\\Health care providers\\IDI-Midwife-Deputy In charge >](95069826-e33d-4365-91d6-3deeba72c532) - § 1 reference coded [1.92% Coverage]

Reference 1 - 1.92% Coverage

R I don’t know of the challenges but what I can say is that when we send the form to the OPD team, they do have issues with the OPD number and maybe it is not well written.

[<Internals\\Health care providers\\IDI-head of finance>](18b0e2f5-3dfc-4320-b9d6-3deebac1e6da) - § 3 references coded [7.15% Coverage]

Reference 1 - 2.50% Coverage

I: What are the challenges you face in claims submission

Resp: We have inadequate staff seriously, here most of our NHIS claim staff are national service personnel and casual workers. Every year they are supposed to leave and you need other hands to train them again (.

Int: How does it affect claim submission?

Resp: It delays and it also sometimes increases the level of mistakes because they are not well qualified staff. Most of them did accounting and have no knowledge about pharmacy and health system.

Reference 3 - 1.80% Coverage

Int: Please what are some of the reasons why the reject some of the claims?

Resp: Sometimes poor handwriting and wrong prescription against diagnosis.

[<Internals\\Health care providers\\IDI-midwife->](c282ffa7-31c8-4cf1-8fd6-3deebae80c64) - § 1 reference coded [3.13% Coverage]

Reference 1 - 3.13% Coverage

**Res: Yes, sometimes you take it to them and then they tell you this particular ailment does not go with this drug and this drug is been given overdose and they won’t pay that kind of issues normally arises (IDI39 yr old midwife-Ajumako)**

[<Internals\\Health care providers\\IDI-Accountant->](dfd23f0f-4df6-4615-82d6-3deebb1559a1) - § 2 references coded [4.05% Coverage]

Reference 1 - 1.89% Coverage

Int: yes errm we are using a software so the challenges may be, so the last challenge we had recently or tackled was health insurance changed the form of submission. And the software owner also had to do some changes but he charged us. We had to pay upfront before he sent somebody from Accra to come and do that. So finally they came to do that before we sent the submissions.

Reference 2 - 2.16% Coverage

Voice: it caused because we knew of the old way of submission. NHIS told us that well we even sent some of the claims and they returned them and said they have changed their way of submission or the form in which they use to submit. So we had to work on them and bring them back. And because the software provider needs money, it took us sometime. Before we gave the money and they came to work on it. so it really delay sometime.

[<Internals\\Health care providers\\IDI-Deputy Chief Health Adminstrator->](78faf6d3-b67b-41d9-8fd6-3deebb404497) - § 1 reference coded [6.64% Coverage]

Reference 1 - 6.64% Coverage

I What has been some of the challenges with claim system?

R Yes before we used to submit at the district level and the cost was fast and less in vetting and they could alert you when there are genuine errors in the claims then you do corrections. But for the processing center, thus the CPC, due to the high volumes of work they will not have the time to call you to come and address genuine errors on the claim sheets. They will go ahead and this causes some deductions. Also, because it is not at the district level, when there are errors, it takes time before you get to know about it. When it was at the district level, they could glance through and if there are errors, they will call you to correct it quickly but at the CPC, it could take like 8 months before issues are brought to your attention. So, these are some of the issues that we face as far as the claims submission is concerned.

[<Internals\\Health care providers\\IDI- Medical Superintendent ->](fe3c554a-3bb9-463f-a7d6-3deebb68cd65) - § 1 reference coded [5.48% Coverage]

Reference 1 - 5.48% Coverage

I What has been some of the challenges with the claim submissions?

R It depends on the arrangement of the hospital management because we don’t have a grade on the Ghana Health Service scheme for employing staff to work on NHIS claims. So those working on the claims now are casuals that we have employ and we need to provide them with the logistics to work and submit within the two weeks period. If there are no logistics to work with, then claims could be left there for three months not yet submitted.

[<Internals\\Health care providers\\IDI-Medical Superintendent >](6ca5c3aa-3208-4bf4-9ed6-3deebb9155f8) - § 1 reference coded [2.18% Coverage]

Reference 1 - 2.18% Coverage

I What has been some of the challenges with the claim submissions?

R Don’t have any challenge with that and we try to submit within a month.

[<Internals\\Health care providers\\IDI-Hospital administrator->](3c7fac7e-97f5-40fe-91d6-3deebbc10564) - § 2 references coded [3.64% Coverage]

Reference 1 - 2.53% Coverage

Resp: it’s the HR it’s the HR and to some extent the equipment because many a times you are working with a desk top and the light will go off and there are issues here and there you have to quickly solve it because you are on a time bomb. The circular that I was talking about its like if you submit your claims late, like about 3months don’t expect to get reimbursement when people are getting reimbursement for that month so we do our best to ensure that you know NHIS holds this hospital, herr, if you lose guard you will be left out.. yeah

Reference 2 - 1.11% Coverage

Resp: no, no the CPC office at Cape Coast they are cool since we have (fumbles) the personnel have been on duty for a while, the NSS year began this month so we have 3 people we are training on the input. mhmmm. Basically we are on track

[<Internals\\Health care providers\\IDI- Deputy Chief Accountant->](51d2e908-a05b-49b2-81d6-3deebbec1627) - § 2 references coded [6.02% Coverage]

Reference 1 - 3.50% Coverage

I What has been some of the challenges with claim system?

R You know we see a lot of people and you have to do these things manually and its so cumbersome. There are lots of human errors and duplication of figures. Also, we don’t have the numbers in terms of staff to do that so we are happy when we get lots of service personnel and we push them there to handle that.

Reference 2 - 2.52% Coverage

R Its not easy when the service personnel leave though few still hang around and these days we have been told not to employ any casuals so we use the service personnel a lot. At times we need to motivate them to come and do additional duties and that is what we do.

[<Internals\\Health care providers\\IDI-Deputy Chief Pharmacist->](0274cbfb-ba52-4503-aed6-3deebc2ed937) - § 1 reference coded [3.05% Coverage]

Reference 1 - 3.05% Coverage

R First when we were submitting claims manually we used to have challenges but now we have migrated to E claims. But we still use the claim forms and its entered into the computer and we send both soft and hard copies and the challenges have reduced. The challenge is that we don’t get the paper to use to print the claims and it delays.

[<Internals\\Health care providers\\IDI- Health Service administrator->](bc053ab0-d4f7-4e92-9ed6-3deebc57616b) - § 2 references coded [4.25% Coverage]

Reference 1 - 2.59% Coverage

R No, they are owing us over 900, 000 cedis after some deductions made. When we started with the e-claim, they were paying us everything then some five months later, they started retaining 10% of all submission and claim that they have not done the vetting and after the vetting, you might not get 100%. So, when you submit claims they pay only 90%.

Reference 2 - 1.66% Coverage

I After vetting do they pay the 10% withheld?

R We don’t get any feedback that is why we have calculated and they owe us 900, 000 cedis. We are not sure they will pay but they said they will pay after the vetting process.

[<Internals\\Health care providers\\IDI-Medical Superintendent->](303021d4-6193-44b3-91d6-3deebc824cc9) - § 1 reference coded [4.73% Coverage]

Reference 1 - 4.73% Coverage

R Government is not employing staff to do the claim submission so we have to get people to do and pay them at the cost of the facility. Also, the facility submits claims of about 300 000 cedis monthly and we see thousands of clients. We have national service persons who help with that but after service they have to go and we have to train new people who don’t know the diagnosis. Ideally, we need to have medical people but can’t get doctors doing those claims submissions. Also, the national service persons cannot see the handwriting of the prescribers and end up making mistakes. We also train the doctors but new batch keeps coming in and it’s not easy. Some facilities have less figures but we have a lot and we end up using our resources and staff to handle this NHIS claim submission.

[<Internals\\Health care providers\\IDI- Maternity in charge->](1318a536-efbe-46e9-a3d6-3deebcaad598) - § 1 reference coded [3.85% Coverage]

Reference 1 - 3.85% Coverage

I What has been some of the challenges with claim system?

R In our part we write all the drugs needed by the client and when it gets to the dispensary then they will enter all that cost and then send it so there isn’t much challenges.

[<Internals\\Health care providers\\IDI- Medical Sup In-charge>](0d23e9f1-c823-4c39-a6d6-3deebcd35d27) - § 1 reference coded [3.12% Coverage]

Reference 1 - 3.12% Coverage

R Sometimes we have problems with the computer we use to send in the claims and the computer keeps breaking down and don’t have the funds to pay for repairs and we keep changing repairers because we are not able to pay them after fixing the computer for us. We are short of money to undertake any activity

[<Internals\\Health care providers\\IDI- Deputy Director of Nursing Services->](44d0b877-60dc-446e-96d6-3deebd236d27) - § 1 reference coded [3.77% Coverage]

Reference 1 - 3.77% Coverage

I What has been some of the challenges with the claim submissions in the facilities that you supervise?

R Some of them don’t use the right generic names during the inputting phase and at times the diagnosis will be wrongly inputted. Also, the handwritings of the prescribers cannot be seen well. Also not submitting at the right stipulated time.

[<Internals\\Health care providers\\IDI-Medical Direector>](fd1ef618-af77-4b1b-acd6-3deebd4730dd) - § 1 reference coded [2.19% Coverage]

Reference 1 - 2.19% Coverage

I What has been some of the challenges with the submissions?

R No, don’t have challenges.
