# Supplementary material for: How does it affect service delivery under the National Health Insurance Scheme in Ghana? Health providers and insurance managers perspective on submission and reimbursement of claims
Source: PLoS One. 2021 Mar 2;16(3):e0247397. doi: 10.1371/journal.pone.0247397 (PMC7924798; doi:10.1371/journal.pone.0247397)
Supplement: S2 File — (ZIP) [file pone.0247397.s002.zip › S1 File. Study aata/Health providers and Managers/Delay affect service delivery.docx]

[<Internals\\Health care providers\\IDI-Facility In-charge->](c600cba7-bc3d-41c8-8fd6-3deeb9ce411c) - § 1 reference coded [4.16% Coverage]

Reference 1 - 4.16% Coverage

R The NHIS should re-imburse the facilities on time and they should put in place measures to work on submitted claims on time to able to improve on service delivery always.

[<Internals\\Health care providers\\IDI-Facility In-charge>](42a21635-e6b2-4991-a5d6-3deeba398cc3) - § 1 reference coded [2.87% Coverage]

Reference 1 - 2.87% Coverage

R Yes because suppose we are to render account, how are we able to do that to report the drugs taken from them and how much has been paid to the hospital. So we don’t know how much we owe them and how much they also owe us.

[<Internals\\Health care providers\\IDI-Midwife-Facility Deputy In-charge ->](95069826-e33d-4365-91d6-3deeba72c532) - § 1 reference coded [2.13% Coverage]

Reference 1 - 2.13% Coverage

I How has these experiences affected service delivery?

R For here we don’t collect monies at the OPD and consulting but we do get our drugs from the Health center and we care for patients.

[<Internals\\Health care providers\\IDI- staff midwife->](4b08f388-f3dd-4208-82d6-3deeba98ebe5) - § 1 reference coded [5.26% Coverage]

Reference 1 - 5.26% Coverage

I How is the treatment given to clients irrespective of the delays in reimbursement?

R For that one I will say that clients are well treated and they do appreciate it and thank us a lot. We treat everyone equally.

[<Internals\\Health care providers\\IDI- head of finance->](18b0e2f5-3dfc-4320-b9d6-3deebac1e6da) - § 1 reference coded [3.35% Coverage]

Reference 1 - 3.35% Coverage

Int: How has this affected your services you provide here?

R: If the payment delays, we unable to pay our suppliers, who also find it difficult to supply our drugs which leads to shortage of drugs. It also delays the payment our casual workers and a hungry man is an angry man.

[<Internals\\Health care providers\\IDI- midwife->](c282ffa7-31c8-4cf1-8fd6-3deebae80c64) - § 2 references coded [6.25% Coverage]

Reference 1 - 2.72% Coverage

Res: Not really. Sometimes the hospital buys and then still we give it to them, they buy on credit so anytime the NHIS pays, and we just pay those that they bought the items from.

Reference 2 - 3.54% Coverage

Int: Has it affected the facility?

Res: Not much, we still provide them with whatever we are to provide. There has never been any instances that NHIS has paid that we refuse to give our clients any treatment. We normally treat them.

[<Internals\\Health care providers\\IDI-Hospital Accountant->](dfd23f0f-4df6-4615-82d6-3deebb1559a1) - § 1 reference coded [4.62% Coverage]

Reference 1 - 4.62% Coverage

Voice: you end up writing to buy consumables. You end up writing to buy reagents, you end up writing for patients to go and buy certain things and come. Like the drugs, if medical stores they can give us credit and they don’t have and you are owing the other outside suppliers much, so much that if you don’t pay and there is no reimbursement. They will not be willing to give you. And the same thing as this errr the non-drug consumables. If the supplier has supplied a big quantity, a large quantity and you have not paid, the supplier will not be willing to give you. So you end up putting pen on paper for the client to go and do certain things in order to deliver the service. It in a certain way delays or it doesn’t give quick response to the patient, because the patient has to go out. Maybe whether he has money or he doesn’t have money he has to go and look for certain thing and come back. So it is a problem.

[<Internals\\Health care providers\\IDI- Deputy Chief Health Adminstrator->](78faf6d3-b67b-41d9-8fd6-3deebb404497) - § 1 reference coded [5.35% Coverage]

Reference 1 - 5.35% Coverage

I How has these experiences affected service delivery?

R If insurance delays, it will be difficult to pay our suppliers and casuals. Now government don’t engage labourers and we pay them from our IGF so if insurance delays then its difficult to pay them and also take care of other administrative costs.

We need to buy fuel for generators and for the cars. Those without insurance and come for cash and carry services are very small thus Only 10%. So if NHIS delays to reimburse us it affects service delivery especially at the administrative side and suppliers will be chasing you as well. If you don’t pay suppliers they cannot supply you with consumables and that affects service delivery as well and quality of service to clients.

[<Internals\\Health care providers\\IDI-Medical Superintendent ->](fe3c554a-3bb9-463f-a7d6-3deebb68cd65) - § 1 reference coded [6.77% Coverage]

Reference 1 - 6.77% Coverage

R It has affected so much because you need money to run the service and also purchase drug and consumables and when you are buying the items on credit, we tell the suppliers that we will pay within the 90 days period but even after a year we are not able to pay them. So sometimes we need to be nice to them and inform them that once insurance reimburses then you will pay them. In that case you have to be very wise in using the available items efficiently. Sometimes you have shortage of drugs and consumables and the suppliers don’t want to come for the tender because when they supply the items they are not paid on time.

[<Internals\\Health care providers\\IDI- Medical Superintendent of Hospital>](6ca5c3aa-3208-4bf4-9ed6-3deebb9155f8) - § 1 reference coded [14.89% Coverage]

Reference 1 - 14.89% Coverage

I How has these experiences affected service delivery?

R It has affected us because you are not able to replace equipment as quickly as possible because we see 150 patients a day and you need to replace equipment that you use regularly, also light, consumables and more expensive tools like the autoclave which breaks down and need to be replaced but don’t have the money to do that. It cost like 7,000 and you need to use your own money before if government will replace it. Because of all these activities you are always cash trapped. Since 2009 there has been a freeze on recruitment of para medical staff. Like cleaners, drivers, security so the hospital employs and pay as casuals and you need to pay them and also fix broken down equipment. Also NHIS funds delay in coming and it affects service delivery. Luckily for us the suppliers understand the system and its quite stressful. Some do cut you off but we keep things going and its quite stressful some times.

[<Internals\\Health care providers\\IDI-Health Service Administrator->](3c7fac7e-97f5-40fe-91d6-3deebbc10564) - § 1 reference coded [5.93% Coverage]

Reference 1 - 5.93% Coverage

Resp: Hmmm my sister! (Chuckles) sometimes errr I feel so bad, I feel so bad that a little intervention could have saved somebody’s life err we have challenges, we have serious challenges, just as I mentioned earlier creditors disturbing you hei my money and by law the day a creditor will submit his items to you, you have 90 days to pay back the item. And now it goes beyond the 90 days. Challenges dea it is enormous, its enormous, I wish you were here sometime last month just taking you to the casualty and to the children’s ward. People were lying on the floor. People were sitting there because we can’t turn them away. Never we will never do that here because I mean we … when you go to the children’s ward they were sharing. We have advocated for this ward for so many years but no one has come to our aid.. and if NHIS wa s good and building a the government of the day is also on a good level, I think building a children’s ward would go a long way. I have talked to some people. Sons of this town but it are like they are not forth coming. We’ve written letters to NGOs but still it is …. So with NHIS payment and the challenges, your guess is as good as mine. it hinders excellent service delivery. It hinders excellent service delivery. So that is that.

[<Internals\\Health care providers\\IDI-Deputy Chief Accountant->](51d2e908-a05b-49b2-81d6-3deebbec1627) - § 1 reference coded [1.60% Coverage]

Reference 1 - 1.60% Coverage

R It has affected us a lot. All those calling me are suppliers whom we owe as far as 2016 and you can’t blame them. They need their monies because they are in business.

[<Internals\\Health care providers\\IDI- Deputy Chief Pharmacist->](0274cbfb-ba52-4503-aed6-3deebc2ed937) - § 1 reference coded [2.44% Coverage]

Reference 1 - 2.44% Coverage

R A lot, our gutters are chocked and infections all over and its affecting us alot. The place is not neat at it used to be. In terms of service delivery and attendance has reduced and the staff is not giving their best because the place is not friendly as it was to be.

[<Internals\\Health care providers\\IDI- Health Service administrator->](bc053ab0-d4f7-4e92-9ed6-3deebc57616b) - § 1 reference coded [1.50% Coverage]

Reference 1 - 1.50% Coverage

I How has these experiences affected service delivery?

R first, we are unable to purchase some items we need and some are medical equipment, administrative and paying of allowance to some of the staff.

[<Internals\\Health care providers\\IDI- Medical Superintendent->](303021d4-6193-44b3-91d6-3deebc824cc9) - § 1 reference coded [2.77% Coverage]

Reference 1 - 2.77% Coverage

I How has these experiences affected service delivery?

R It has improved services and I will say that NHIS has improved Ghana’s health system to a larger extent. The money is not coming and we cannot do more and we cannot expand. You go down and realize that EnT is being done in 2 rooms, psychiatry in one room, eye has one room with optometrist and nurses sitting around the same desk. But with NHIS money, we could have expanded as we were doing in the past.

[<Internals\\Health care providers\\IDI- Maternity in charge->](1318a536-efbe-46e9-a3d6-3deebcaad598) - § 1 reference coded [3.77% Coverage]

Reference 1 - 3.77% Coverage

I How has these experiences affected service delivery?

R Yes so much because when the suppliers get their money on time, they are able to supply us early and we care for the clients so when it delays, it affects service delivery.

[<Internals\\Health care providers\\IDI- Medical Sup In-charge>](0d23e9f1-c823-4c39-a6d6-3deebcd35d27) - § 1 reference coded [2.58% Coverage]

Reference 1 - 2.58% Coverage

R Affected us a lot and we have to be improvising. We are not able to contact the suppliers because we have not paid them for items previously supplied. Even now we do photocopies of documents for reuse since we don’t have the money to purchase new ones.

[<Internals\\Health care providers\\IDI-Deputy Director of Nursing Services->](44d0b877-60dc-446e-96d6-3deebd236d27) - § 2 references coded [6.66% Coverage]

Reference 1 - 2.96% Coverage

I How has these experiences affected service delivery?

R Yes because these monies are supposed to be used as reimbursement for services rendered so if these monies are not released, then it will be difficult to make purchases and also to render quality services needed.

Reference 2 - 3.70% Coverage

I Has that necessitated some of the co-payments measures in some facilities?

R We do not have such issues reported yet but thinking aloud, it could happen and even though they work under us most of the things that happen there we don’t know. But with our facilities because we work with them we get to know early what is going on there.
