# Supplementary material for: How does it affect service delivery under the National Health Insurance Scheme in Ghana? Health providers and insurance managers perspective on submission and reimbursement of claims
Source: PLoS One. 2021 Mar 2;16(3):e0247397. doi: 10.1371/journal.pone.0247397 (PMC7924798; doi:10.1371/journal.pone.0247397)
Supplement: S2 File — (ZIP) [file pone.0247397.s002.zip › S1 File. Study aata/Health providers and Managers/Experiences on submission and reimbursement of claims.docx]

[<Internals\\Health care providers\\IDI- Facility In-charge>](42a21635-e6b2-4991-a5d6-3deeba398cc3) - § 2 references coded [3.92% Coverage]

Reference 1 - 2.77% Coverage

I What has been some of the experiences for the reimbursement of claims?

R The experience is that we don’t get feedbacks to know what is happening.

I Have you had issues with delay of reimbursement of funds?

R No

Reference 2 - 1.15% Coverage

I Do they tell you whether insurance has paid or not?

R No, they don’t tell us anything.

[<Internals\\Health care providers\\IDI- Midwife-Deputy In-charge ->](95069826-e33d-4365-91d6-3deeba72c532) - § 1 reference coded [2.09% Coverage]

Reference 1 - 2.09% Coverage

R No we cant tell and even if there are no drugs at the Asuofia Health center, they will call and inform us about that so then you will write it for the client to go and buy it in town.

[<Internals\\Health care providers\\IDI- Staff midwife->](4b08f388-f3dd-4208-82d6-3deeba98ebe5) - § 1 reference coded [4.65% Coverage]

Reference 1 - 4.65% Coverage

I Does NHIS delay in claims reimbursements to the facility?

R For that one I have not followed up to know the process it takes for reimbursements and if there are any delays I cannot tell.

[<Internals\\Health care providers\\IDI-head of finance->](18b0e2f5-3dfc-4320-b9d6-3deebac1e6da) - § 3 references coded [9.07% Coverage]

Reference 1 - 1.80% Coverage

Int: Please what are some of the reasons why the reject some of the claims?

Resp: Sometimes poor handwriting and wrong prescription against diagnosis.

Reference 2 - 3.41% Coverage

Int: Please I want you to share with me some of your experiences in receiving reimbursement?

Resp: Actually it delays unnecessary, as am talking to you we are in September and the last one we received was in late December 2017. So late payment and the amount of rejection is also high.

Reference 3 - 3.86% Coverage

Resp: With the reimbursement, the drug component and service component are paid together into one account and I think it not the best. If they pay everything into the service account there is a lot of pressure on the service account. So management may use some drug money for services activities whereby it affects the drug.

[<Internals\\Health care providers\\IDI- Facility Midwife->](c282ffa7-31c8-4cf1-8fd6-3deebae80c64) - § 2 references coded [11.75% Coverage]

Reference 1 - 1.59% Coverage

Int: If they would have to pay, do they normally pay in full?

Res: They pay part not full, not everthing

Reference 2 - 10.15% Coverage

Res: Sometimes when they come going for the insurance it takes a longer time, sometimes they get there and they tell you there is a shortage of parts that they have to give to their clients. They have to come, they don’t have money and they don’t have the NHIS. Sometimes the claims should be paid on time so that at least we can purchase the items that we need to work with because if we don’t have enough we might turn up taking care of them but it will just be a little bit lower. Because we don’t have enough money to buy the items you ask them to go and buy Fanta they will go for consulting alright but with the drugs they would have to go outside and buy them.

[<Internals\\Health care providers\\IDI-Facility Accountant>](dfd23f0f-4df6-4615-82d6-3deebb1559a1) - § 1 reference coded [2.55% Coverage]

Reference 1 - 2.55% Coverage

Voice: sometimes they jump. They will jump some of the monies and pay current monies so if you don’t do follow up those monies would have locked there. They will never come. Assuming the money to pay claims, or the money to sometimes I don’t know what they sometimes want. They want to pay claims for this year and they lock up last year. Even the last year one they pay this year and if they pay like that across board the perception is that they have paid up to this year. Meanwhile there is still lock up.

[<Internals\\Health care providers\\IDI-Deputy Chief Health Service Adminstrator->](78faf6d3-b67b-41d9-8fd6-3deebb404497) - § 2

I What has been some of the experiences for the reimbursement of claims?

R Previously the delays are there and like I said we are having three months that are overdue but now its better because previously we could have over 8 to 13 months overdue but if its now 5 months overdue, I will say that there has been improvement. But another difficulty is that they do some deductions and you don’t get the reasons for those deductions and the monies are transferred to your account directly. Previously we picked the cheques at the district office and if you are due say 10 cedis and they pay 9 cedis you are given the reasons why. But now the payments are made from the central source and the reasons for deductions will not be known now and the refund will be done at a later date though it will take time.

Reference 2 - 8.97% Coverage

R What I will say is that insurance is now the life blood for health delivery, life blood for facilities and for the clients so the NHIA should be proactive in ensuring the sustainability of the health insurance. They should ensure frequent reimbursement and also review the tariffs. At times over three years and the tariffs have not been reviewed and that affects service delivery. In Ghana every year there are increment in prices of goods and services and insurance will not review most of the tariffs and it becomes difficult to get the consumables. For example if insurance is reimbursing me for 5 cedis when I bought it for 6 cedis and it becomes difficult. We request that the review should be done every year to commensurate the cost of living in the country. The central system of claims submissions delays so if they can equip the district to take back the mandate of claims management at the district level it will be better. Because we need to come to Kumasi and join queue whiles the district office is there. So NHIA should reconsider this issue again and come out with something substantial to minimize the challenges and burden on the only CPS here in Kumasi and it will be better. Thank you.

[<Internals\\Health care providers\\IDI- Medical Superintendent>](6ca5c3aa-3208-4bf4-9ed6-3deebb9155f8) - § 1 reference coded [2.32% Coverage]

Reference 1 - 2.32% Coverage

R Its in full. When you submit, they vet the claims and then when there are discrepancies, then they correct them with you and pay you the amount due.

[<Internals\\Health care providers\\IDI- Health Service Administrator->](3c7fac7e-97f5-40fe-91d6-3deebbc10564) - § 2 references coded [6.83% Coverage]

Reference 1 - 5.90% Coverage

R: the claims reimbursement is a bit better than before. That one truth has to be said. I don’t want to sound political or anything. I am speaking from the perspective of a hospital administrator. Because before then my sister it wasn’t good. it wasn’t good at all but now it is better. The systems were not running. We were just living by grace. Sometimes because of the delays in the claims reimbursement nu. The creditors they also start playing this thing with you. The thing is 5 cedis because he knows his money will delay he will make it 10 cedis. And you won’t get it anywhere. No one wll give it to you on credit so what will you do. Even if it is genuine and it is authentic no problem but sometimes they bring you inferior goods. And they know the money will take long. must duly be given. And if there are any powers that be that can help Ajumako District Hospital with a children’s ward we would be so so so grateful so please be our voice. Be our advocate out there. Ajumako District Hospital needs a children;s ward seriously!, seriously. We have a dedicated children’s ward. That is the one at the last end but the we have a male and female ward so when we get the children’s ward we will move the children and give one ward to the males and one ward to the females.

Reference 2 - 0.93% Coverage

Int: soin what year did it start?

Resp: err last year, last year, last year just around this time, just around this time then it started improving. Just around this time , then it started improving.

[<Internals\\Health care providers\\IDI- Deputy Chief Accountant- >](51d2e908-a05b-49b2-81d6-3deebbec1627) - § 1 reference coded [1.58% Coverage]

Reference 1 - 1.58% Coverage

R They don’t come on schedule and it could take like three months and you have not received any reimbursement. But now there has been improvement as it come monthly.

[<Internals\\Health care providers\\IDI- Deputy Chief Pharmacist- >](0274cbfb-ba52-4503-aed6-3deebc2ed937) - § 1 reference coded [6.12% Coverage]

Reference 1 - 6.12% Coverage

R Because of the delays we are not able to give out our best at all. And we are working under very bad conditions. Those days after injecting someone, you throw both syringe and needle away but now we throw just the needle and reuse the syringe because there is no money to replace it. It cannot be replaced now because there is no money to buy them and the pharmacy tried to buy them now. For the gloves, we are blessed in a way because this is an HIV center and the test kits comes with gloves and we use those gloves beucase there is no money to buy new ones. There was times when we used gloves severally but we don’t have and its having effects on our service delivery.

[<Internals\\Health care providers\\IDI- Health Service administrator->](bc053ab0-d4f7-4e92-9ed6-3deebc57616b) - § 1 reference coded [4.06% Coverage]

Reference 1 - 4.06% Coverage

R The delays in reimbursement of claims affect our care in terms of purchase of things to cater for the patients. Even though we go to the market to get items through tender process, but you get suppliers calling asking when they will be paid and this is on daily basis. Secondly, we are not able to make purchase of equipment and consumables because we owe and people cannot give you more when you have not settled the old dept. Also there are minor things to do. Even though we generate income internally, the bulk is from NHIS thus 84% and the 16% is cash and carry.

[<Internals\\Health care providers\\IDI- Medical Superintendent->](303021d4-6193-44b3-91d6-3deebc824cc9) - § 1 reference coded [13.63% Coverage]

Reference 1 - 13.63% Coverage

I What has been some of the experiences for the reimbursement of claims?

R In the past it was run in a mutual district system and we send our returns to Bantama and they could come over to see our folders. We got our reimbursement within two months and things were ok until processing claims moved to Accra and cheques were paid from there. So those in district and regions are useless and over ten months no payment received. After a while, they paid few months and left the other month and published in the dailies that they had paid. So creditors will be following us for their monies and at a point in time we had to start charging patients for services like the lab, x-ray because we had used all our capital**.**

We then started asking client to do the top-ups and all these made us go through a lot. This persisted till they introduced capitation. This was good to us but not to the general public due to the health seeking behaviors. If someone was sick, he or she can decide where to go but it was not good to the general public because they were restricted but for us the number you treated were given to us. So we were given a population of 17 000 to take care of and our numbers were reducing but we were not worried because we were given the money for that population. So sometimes the number reduces and it never went up and we had the funds to operate with. Then it stopped and you end up treating patients and the funds comes at the end of another month which of cause is no more capitation. Then we joined the chorus that the capitation was not good and it stopped. Now we got back to the old system and that affected us. Though patients didn’t have problems because they were taken care off, we suffered as a facility. Our numbers shot up and we used the little money we had to purchase consumables and that is the situation. More people patronize the place and we are not getting any reimbursement. So it is the money that we get from co-payment that we use to run the facility, pay casuals and buy bulbs, bed sheet, detergents and now we are living from hand to mouth.

When we have doctors on leave, we need to get the locum doctors to support and as a big a facility we need more doctors around always. If we had money we could employ more staff to assist.

[<Internals\\Health care providers\\IDI- Maternity in charge->](1318a536-efbe-46e9-a3d6-3deebcaad598) - § 1 reference coded [3.47% Coverage]

Reference 1 - 3.47% Coverage

I What has been some of the experiences for the reimbursement of claims?

R If it is submitted, it comes in the advancement of the care and I don’t know whether it delays or not and all that rests on management.

[<Internals\\Health care providers\\IDI- Deputy Director of Nursing Services->](44d0b877-60dc-446e-96d6-3deebd236d27) - § 1 reference coded [3.07% Coverage]

Reference 1 - 3.07% Coverage

I What has been some of the experiences for the reimbursement of claims?

R After the vetting, instead of them writing report to discuss with the facility, they won’t do it and those they don’t think of reimbursing they will remove them and when your money comes it will be less.

[<Internals\\Health care providers\\IDI-Medical Direector,>](fd1ef618-af77-4b1b-acd6-3deebd4730dd) - § 1 reference coded [3.31% Coverage]

Reference 1 - 3.31% Coverage

R Like I said earlier on it used to be there were we had delays in reimbursement but now we don’t have those delays and so far so good.
