# Supplementary material for: How does it affect service delivery under the National Health Insurance Scheme in Ghana? Health providers and insurance managers perspective on submission and reimbursement of claims
Source: PLoS One. 2021 Mar 2;16(3):e0247397. doi: 10.1371/journal.pone.0247397 (PMC7924798; doi:10.1371/journal.pone.0247397)
Supplement: S2 File — (ZIP) [file pone.0247397.s002.zip › S1 File. Study aata/Health providers and Managers/Guidelines for submission claims.docx]

[<Internals\\Health care providers\\IDI- Facility In-charge>](42a21635-e6b2-4991-a5d6-3deeba398cc3) - § 1 reference coded [3.32% Coverage]

Reference 1 - 3.32% Coverage

I what are some of the guidelines that cover for claim submissions? What are some of them?

R They educated us on how to complete the claim forms and then after that you are to submit on the 5th day of the new month and they will do the entry and submission.

[<Internals\\Health care providers\\IDI- Midwife-Deputy In charge ->](95069826-e33d-4365-91d6-3deeba72c532) - § 1 reference coded [2.54% Coverage]

Reference 1 - 2.54% Coverage

R I don’t know anything about the claims but the pharmacy fills the claims and send to Asuofia Health center.

. I know they check the date, the condition the patient came with, whether inpatient or outpatient and the NHIS number (.

[<Internals\\Health care providers\\IDI- Staff midwife->](4b08f388-f3dd-4208-82d6-3deeba98ebe5) - § 1 reference coded [2.41% Coverage]

Reference 1 - 2.41% Coverage

I Do you know about the guidelines for claim submission for NHIS?

R No please don’t have an idea.

[<Internals\\Health care providers\\IDI- head of finance->](18b0e2f5-3dfc-4320-b9d6-3deebac1e6da) - § 2 references coded [7.03% Coverage]

Reference 1 - 5.23% Coverage

Resp: I have a bit, what I have observed is that at the end of the day, we have an officer who compile all their documents, key them in the system then at the end of the month we vet them, but most of the time we are not able to complete the on the last day of the month so it extends to the following month. So sometimes in the second week of the following month it ready then we submit it to the claims processing unit at central region.

Reference 2 - 1.80% Coverage

Int: Please what are some of the reasons why the reject some of the claims?

Resp: Sometimes poor handwriting and wrong prescription against diagnosis.

[<Internals\\Health care providers\\IDI-Midwife->](c282ffa7-31c8-4cf1-8fd6-3deebae80c64) - § 1 reference coded [4.30% Coverage]

Reference 1 - 4.30% Coverage

Res: When you come and you fill everything, and then you just submit to the claims unit make sure whatever you are required to fill you fill it correctly, then you have the claims office so they work on it before they take it back to the insurance office for validation and the rest.

[<Internals\\Health care providers\\IDI- Deputy Chief Health Adminstrator->](78faf6d3-b67b-41d9-8fd6-3deebb404497) - § 1 reference coded [1.69% Coverage]

Reference 1 - 1.69% Coverage

R So submit claims on the 14th of the following month.

I Do you use e-claims for your facility?

R For now no but we have a software where we submit our claims to the CPC. We don’t use the e-claims but send through hard copies.

[<Internals\\Health care providers\\IDI- Medical Superintendent ->](fe3c554a-3bb9-463f-a7d6-3deebb68cd65) - § 1 reference coded [1.95% Coverage]

Reference 1 - 1.95% Coverage

R Claims are to be submitted by the second week of the ensuing month. And it takes one month for vetting and then three months for reimbursement which take long for some time now.

[<Internals\\Health care providers\\IDI-Medical Superintendent of Hospita>](6ca5c3aa-3208-4bf4-9ed6-3deebb9155f8) - § 1 reference coded [4.93% Coverage]

Reference 1 - 4.93% Coverage

R You aggregate the claims according to the service provided. Then submit it within three months at the end of the service. So the August ones need to be submitted by September and you segregate them by number of inpatient, surgery, laboratory, OPD cases attended to and then submit. So that is the guidelines we follow (.

[<Internals\\Health care providers\\IDI-Administrator->](3c7fac7e-97f5-40fe-91d6-3deebbc10564) - § 3 references coded [7.02% Coverage]

Reference 1 - 3.88% Coverage

R: ideally, we are supposed to submit the previous month’s claims by 5th day of the following month. We are in September, we are supposed to submit August claims by the 5th of September but as I speak we have HR issues so we are still working on it.

Errm NHIS and claim submission and claims reimbursement, you and I know it has its ups and downs but I think currently it is improving. Its improving its improving it’s much better than before. There’s a circular I just saw signed by the NHIS man. I think something Annoh I don’t remember the first name. It’s like every month they will try and make sure they pay I month which will help sustain the health facilities. As at now creditors, I will explain my course. Creditors will be calling you and the creditors even know that the NHIS has paid before you will even know. Yes

Reference 2 - 2.11% Coverage

R: let me show you the process. It goes to CPC, CPB does all the vetting. After the vetting, and they give you a vetting report. Ok its 2 trenches. 2 different accounts. We have the cleric account and the service account. Maybe service you have submitted 77 thousand and drugs maybe you submitted 60 thousand after vetting maybe the 77 becomes 70 and the 66 came to about 58 thousand. So they send you a report explaining why the deduction was made. The final vetted one is then sent to Accra so what Accra. Do you understand? So when they are paying, they can’t pay less than what CPC has submitted.

Reference 3 - 1.03% Coverage

Resp: yes GPC. They will send the final one to Accra so what Accra has is the vetted report. Do you understand? So when they are paying, they can’t pay less than what CPC has submitted. So they don’t short change us here.

[<Internals\\Health care providers\\IDI-Deputy Chief Accountant->](51d2e908-a05b-49b2-81d6-3deebbec1627) - § 1 reference coded [4.18% Coverage]

Reference 1 - 4.18% Coverage

R You have the right to submit your claims within three months thus a quarter. But over here we trying to submit by the 15th of the ensuring month and we have submitted up to June this year. We have difficulties because here it is done manually. We are trying to mechanize it so we have electronic submission. We are in August but we have submitted up till June so we are trying to submit the others every month to meet up to avoid arrears.

[<Internals\\Health care providers\\IDI- Deputy Chief Pharmacist->](0274cbfb-ba52-4503-aed6-3deebc2ed937) - § 1 reference coded [1.73% Coverage]

Reference 1 - 1.73% Coverage

R As the patient comes for the drugs, we have a claim sheet that they fill then we send to administrator and someone working on the insurance will work on all and submit within a time frame.

[<Internals\\Health care providers\\IDI-Health Service administrator->](bc053ab0-d4f7-4e92-9ed6-3deebc57616b) - § 1 reference coded [3.08% Coverage]

Reference 1 - 3.08% Coverage

R We are supposed to submit claims when the month ends and by the 15th of the ensuing month but then we are doing it electronically so once we key in it goes to Accra directly. But those who are not using the electronic system must submit it two weeks after the month ends. I learnt some facilities delay in sending because it is done manually and they have to sit down and vet the claims before submitting it.

[<Internals\\Health care providers\\IDI- Medical Superintendent->](303021d4-6193-44b3-91d6-3deebc824cc9) - § 1 reference coded [1.55% Coverage]

Reference 1 - 1.55% Coverage

I What are the guidelines that cover for claim submissions?

R That you need to submit on time and it should be within three months and if you delay you need to come and defend it, it also needs to be clean and verifiable and calculations need to be correct.

[<Internals\\Health care providers\\IDI- Maternity in-charge->](1318a536-efbe-46e9-a3d6-3deebcaad598) - § 1 reference coded [3.10% Coverage]

Reference 1 - 3.10% Coverage

R When they come and we fill the claim sheet, it ends at the dispensary and they submit at the NHIS office here and they also work on it and submit to regional level and then national level.

[<Internals\\Health care providers\\IDI-Medical Sup In charge >](0d23e9f1-c823-4c39-a6d6-3deebcd35d27) - § 1 reference coded [4.92% Coverage]

Reference 1 - 4.92% Coverage

R You are supposed to submit claim within three months. And we submit our claims before time and we don’t submit it late. We used to submit late and they delayed in payment so once the fault was from us we need to submit on time to be reimbursed on time as well.

We have the claims processing officers to work and submit claims on time. We have the team that work on the claims to submit on time and we are using NHIS money to get things working but the reimbursement is not coming.

[<Internals\\Health care providers\\IDI- Deputy Director of Nursing Services->](44d0b877-60dc-446e-96d6-3deebd236d27) - § 1 reference coded [3.10% Coverage]

Reference 1 - 3.10% Coverage

I What are some of the guidelines that cover for claim submissions? What are some of them?

R In order for claims to be paid you need to comply with the standards that they use. You have to use generic names, signs and symptoms for diagnosis and these are the few things that I know.

[<Internals\\Health care providers\\IDI-Medical Direector, Regional Hospital>](fd1ef618-af77-4b1b-acd6-3deebd4730dd) - § 1 reference coded [6.30% Coverage]

Reference 1 - 6.30% Coverage

R We do e-claims so we do our claims regularly. When patients are seen, we fill and submit the claims electronically and it goes straight to Accra for processing and not through CPC. Also by the 5th day of the ensuring month, we are supposed to submit claims.
