# Supplementary material for: How does it affect service delivery under the National Health Insurance Scheme in Ghana? Health providers and insurance managers perspective on submission and reimbursement of claims
Source: PLoS One. 2021 Mar 2;16(3):e0247397. doi: 10.1371/journal.pone.0247397 (PMC7924798; doi:10.1371/journal.pone.0247397)
Supplement: S2 File — (ZIP) [file pone.0247397.s002.zip › S1 File. Study aata/Health providers and Managers/How often reimbusrement received.docx]

[<Internals\\Health care providers\\IDI-In charge Child Welfair Clinic->](c600cba7-bc3d-41c8-8fd6-3deeb9ce411c) - § 1 reference coded [4.79% Coverage]

Reference 1 - 4.79% Coverage

I How often does NHIS reimburse the facility?

R We at the RCH we don’t have information on this aspects since we handle the CWC but if you ask the nurses, lab and insurance team, they can help you.

[<Internals\\Health care providers\\IDI-Facility-In charge >](42a21635-e6b2-4991-a5d6-3deeba398cc3) - § 3 references coded [4.51% Coverage]

Reference 1 - 1.10% Coverage

I How often do you get reimbursement from NHIS?

R We don’t receive any reimbursement.

Reference 2 - 1.13% Coverage

I What do they give you for treatment given to clients?

R They don’t give us anything.

Reference 3 - 2.28% Coverage

I How do you get medicine to treat people?

R We take them from the hospital and they provide us with anything we need to treat our clients so anything we get goes back to them.

[<Internals\\Health care providers\\IDI- Midwife-Deputy In charge ->](95069826-e33d-4365-91d6-3deeba72c532) - § 1 reference coded [2.67% Coverage]

Reference 1 - 2.67% Coverage

I How often do you get reimbursement from NHIS?

R That one I can’t tell because I just fill the claim forms and submit to the Asuofia Health center and all the documents are there. They work on them and also send to Nkawie for processing.

[<Internals\\Health care providers\\IDI- head of finance->](18b0e2f5-3dfc-4320-b9d6-3deebac1e6da) - § 2 references coded [4.60% Coverage]

Reference 1 - 1.91% Coverage

Int: How often do you receive reimbursement for the claims you receive?

Resp: Sometimes once in three months, maybe every quarter we are likely to receive one.

Reference 2 - 2.69% Coverage

Int: And please do you receive in full or in part?

Resp: In full, whatever is agreed on after deducting the rejection we everything in full. There always some that are rejected before the amount is deposited into the account.

[<Internals\\Health care providers\\IDI- Midwife-Ajumako District->](c282ffa7-31c8-4cf1-8fd6-3deebae80c64) - § 2 references coded [4.52% Coverage]

Reference 1 - 2.93% Coverage

Res: It takes time. It can take maybe January, October or maybe September, it takes a longer period for them to pay, for now I learnt it has been coming a little bit earlier than it used to be.

Reference 2 - 1.59% Coverage

Int: If they would have to pay, do they normally pay in full?

Res: They pay part not full, not everthing

[<Internals\\Health care providers\\IDI-Facility Accountant->](dfd23f0f-4df6-4615-82d6-3deebb1559a1) - § 2 references coded [1.95% Coverage]

Reference 1 - 1.39% Coverage

Int: ok so how often do you receive reimbursements for claims submitted?

Voice: as and when money comes.

Int: as and when money comes yes because for now deae we receive… for 2 months now we have not received money. Mmmm Last year dea at least every 2 months we get. that was last year, this year too we received up to 8 …. For two months now we have not received money.

[<Internals\\Health care providers\\IDI-Deputy Chief Health Adminstrator->](78faf6d3-b67b-41d9-8fd6-3deebb404497) - § 1 reference coded [3.14% Coverage]

Reference 1 - 3.14% Coverage

I How often do you get reimbursement from NHIS?

R In recent times its very good. And I can say that they are done right and technically its good. Now my facility has been paid up to December 2017. And in 2018, we have technically submitted from January to July and we are waiting since it takes 90 days for the review. So I will say that 2 to 3 months claims are over due that are yet to be paid and to be it is excellent.

[<Internals\\Health care providers\\IDI- Medical Superintendent ->](fe3c554a-3bb9-463f-a7d6-3deebb68cd65) - § 1 reference coded [3.01% Coverage]

Reference 1 - 3.01% Coverage

R Sometimes it goes into arrears for years and it was just yesterday that we received reimbursement for December 2017. Meaning from January to July 2018, we have not received any reimbursement. They should have paid us up to now for the year 2018 but have not received anything.

[<Internals\\Health care providers\\IDI- Medical Superintendent of Hospital>](6ca5c3aa-3208-4bf4-9ed6-3deebb9155f8) - § 2 references coded [6.56% Coverage]

Reference 1 - 3.52% Coverage

R Its erratic and at times it can delay for like two months but having said that since 2017, at least two months has not passed without getting payment. But as at now we are owed for 8 months now which insurance hasn’t paid for.

Reference 2 - 3.04% Coverage

I Are reimbursement received in full or part?

R Its in full. When you submit, they vet the claims and then when there are discrepancies, then they correct them with you and pay you the amount due.

[<Internals\\Health care providers\\IDI- Health Service Administrator->](3c7fac7e-97f5-40fe-91d6-3deebbc10564) - § 1 reference coded [1.76% Coverage]

Reference 1 - 1.76% Coverage

Resp: are yiu saying currently or. Previously it wasn’t good. Previously you can go for about 3 months no payment but now at least every month there will be something. at least every month there will be something. As I speak now the government has cleared the 2017 debt, so we are now left with the 2018. am speaking for this facility, I don’t know about the rest of the facilities.

[<Internals\\Health care providers\\IDI- Deputy Chief Accountant->](51d2e908-a05b-49b2-81d6-3deebbec1627) - § 1 reference coded [2.15% Coverage]

Reference 1 - 2.15% Coverage

R I will say it is better now because at least every month we are getting one month reimbursement. Only last week we got that of November 2017 and they NHIS still owe us October and December 2017 as well as the whole of 2018.

[<Internals\\Health care providers\\IDI-Deputy Chief Pharmacist->](0274cbfb-ba52-4503-aed6-3deebc2ed937) - § 1 reference coded [1.40% Coverage]

Reference 1 - 1.40% Coverage

I How often do you get reimbursement from NHIS?

R Formerly it used to be every three months but for one and half year now, we have not received any funds.

[<Internals\\Health care providers\\IDI-Health Service administrator->](bc053ab0-d4f7-4e92-9ed6-3deebc57616b) - § 1 reference coded [1.83% Coverage]

Reference 1 - 1.83% Coverage

I How often do you get reimbursement from NHIS?

R About 5 months ago it was a big regular but after that it has not been regular. As at now, NHIS have paid to December 2017 and from January 2018, nothing has been paid and its becoming a problem.

[<Internals\\Health care providers\\IDI- Medical Superintendent->](303021d4-6193-44b3-91d6-3deebc824cc9) - § 1 reference coded [5.69% Coverage]

Reference 1 - 5.69% Coverage

R About 10 months now we have not received reimbursement. NHIA owes us about 2 million cedis and we are just hanging and it is a major headache. We wish it should be paid every three months. We are owing lots of our suppliers and we have to go and beg them for items. We cannot even bid for suppliers because when they supply the items we cannot pay them. Once we get some funds, we try to pay every supplier in bits then they are able to supply us new items. That is why most private hospitals cannot run with the health insurance. No private hospital can run genuinely but we are a government agency and need to provide the needed services. We also have to attend to emergencies without asking for anything like whether you have NHIS or not. At times after all the services provided, the patient dies and who take care of the bills? When NHIS started, it was good and was helping us a lot. We were able to put up buildings but now its so difficult.

[<Internals\\Health care providers\\IDI- Medical Sup In charge of Health Center>](0d23e9f1-c823-4c39-a6d6-3deebcd35d27) - § 1 reference coded [2.12% Coverage]

Reference 1 - 2.12% Coverage

I How often do you get reimbursement from NHIS?

R The last time we received reimbursement was 18 months ago. Thus payment recived for Septer 2016 and one month in 2017. We have mot recived any money for 2018.

[<Internals\\Health care providers\\IDI- Deputy Director of Nursing Services->](44d0b877-60dc-446e-96d6-3deebd236d27) - § 1 reference coded [2.25% Coverage]

Reference 1 - 2.25% Coverage

I How often have they received reimbursement from NHIS?

R Submission should be every three months but the reimbursement is not regular. It could take like 10 months and facilities have not been reimbursed.

[<Internals\\Health care providers\\IDI-Hospital Medical Director->](fd1ef618-af77-4b1b-acd6-3deebd4730dd) - § 2 references coded [6.47% Coverage]

Reference 1 - 3.16% Coverage

I How often do you get reimbursement from NHIS?

R So far they have paid every claims for 2017 and I will say that so far so good.

Reference 2 - 3.31% Coverage

R Like I said earlier on it used to be there were we had delays in reimbursement but now we don’t have those delays and so far so good.
