# Supplementary material for: How does it affect service delivery under the National Health Insurance Scheme in Ghana? Health providers and insurance managers perspective on submission and reimbursement of claims
Source: PLoS One. 2021 Mar 2;16(3):e0247397. doi: 10.1371/journal.pone.0247397 (PMC7924798; doi:10.1371/journal.pone.0247397)
Supplement: S2 File — (ZIP) [file pone.0247397.s002.zip › S1 File. Study aata/Health providers and Managers/Last time facility receive reimbursement.docx]

[<Internals\\Health care providers\\IDI-Facility Midwife->](c282ffa7-31c8-4cf1-8fd6-3deebae80c64) - § 1 reference coded [1.65% Coverage]

Reference 1 - 1.65% Coverage

Int: For this facility, when was the last time you were reimbursed?

Res: I would have to check that for you.

[<Internals\\Health care providers\\IDI-Hospital Accountant->](dfd23f0f-4df6-4615-82d6-3deebb1559a1) - § 4 references coded [1.67% Coverage]

Reference 1 - 0.22% Coverage

Int: that was last year, every 2 months. Ok

Reference 2 - 0.56% Coverage

Voice: that was last year, this year too we received up to 8 …. For two months now we have not received money.

Reference 3 - 0.52% Coverage

Int: ok so please how much do you receive for claims submitted? Do you get full or partial?

Voice: 90%

Reference 4 - 0.37% Coverage

Int: June 2018. But you can’t remember the day

Voice: 9th

Int: 9th June

[<Internals\\Health care providers\\IDI- Deputy Chief Health Adminstrator- >](78faf6d3-b67b-41d9-8fd6-3deebb404497) - § 1 reference coded [0.52% Coverage]

Reference 1 - 0.52% Coverage

I When was the last time you received re-imbursement?

R A month ago.

[<Internals\\Health care providers\\IDI-Health Service Administrator->](3c7fac7e-97f5-40fe-91d6-3deebbc10564) - § 1 reference coded [0.27% Coverage]

Reference 1 - 0.27% Coverage

Int: he last time you were paid was

Resp: December 2017.

[<Internals\\Health care providers\\IDI- Deputy Chief Accountant->](51d2e908-a05b-49b2-81d6-3deebbec1627) - § 1 reference coded [1.05% Coverage]

Reference 1 - 1.05% Coverage

I When was the last time you received funds from NHIS?

R Just last week Thursday and it was for November 2017.

[<Internals\\Health care providers\\IDI-Deputy Chief Pharmacist->](0274cbfb-ba52-4503-aed6-3deebc2ed937) - § 1 reference coded [0.71% Coverage]

Reference 1 - 0.71% Coverage

I since when did you get funds from NHIS?

R I think a year and six months ago

[<Internals\\Health care providers\\IDI- Health Service administrator- >](bc053ab0-d4f7-4e92-9ed6-3deebc57616b) - § 1 reference coded [0.57% Coverage]

Reference 1 - 0.57% Coverage

I When was the last time you received funds from NHIS?

R About 4 months ago.

[<Internals\\Health care providers\\IDI- Medical Superintendent->](303021d4-6193-44b3-91d6-3deebc824cc9) - § 1 reference coded [1.32% Coverage]

Reference 1 - 1.32% Coverage

I When was the last time you received funds from NHIS?

R So the government decided to clear backlog from 2016 thus four months funds were released and we still have 2017 fund to be paid to us in addition to 2018 claims.

[<Internals\\Health care providers\\IDI-Medical Sup In charge of Abuakwa Health Center>](0d23e9f1-c823-4c39-a6d6-3deebcd35d27) - § 1 reference coded [10.13% Coverage]

Reference 1 - 10.13% Coverage

I Does it happen due to delay in reimbursement of funds from NHIS?

**R** No It is not because of the reimbursement of funds even though the NHIS has delayed payments of this facility for 18 months. For the whole of 2016, they have paid for only 1 month. When the capitation was there we didn’t have these problems because we were given funds to use for the facility for a month or two and it helped a lot. But when the capitation was stopped, no money has ever come to the facility for 2018. So we should have stopped patients from coming to the facility but for ethics issues we continue to care

We go and borrow and we are owing the suppliers and they keep calling us severally that they need their money. We need to find resources to use in order to care for clients. We need to run photocopies in order to have sheets to be used in the laboratory. At times we get short of material and even have to use plain A4 sheet to prescribe. So we need the money from NHIS to be able to function smoothly.

[<Internals\\Health care providers\\IDI- Deputy Director of Nursing Services->](44d0b877-60dc-446e-96d6-3deebd236d27) - § 1 reference coded [0.87% Coverage]

Reference 1 - 0.87% Coverage

I When was the last time you received funds from NHIS?

R I don’t have an idea.

[<Internals\\Health care providers\\IDI-Medical Director, Regional Hospital- >](fd1ef618-af77-4b1b-acd6-3deebd4730dd) - § 1 reference coded [3.16% Coverage]

Reference 1 - 3.16% Coverage

I How often do you get reimbursement from NHIS?

R So far they have paid every claims for 2017 and I will say that so far so good.
