# Supplementary material for: How does it affect service delivery under the National Health Insurance Scheme in Ghana? Health providers and insurance managers perspective on submission and reimbursement of claims
Source: PLoS One. 2021 Mar 2;16(3):e0247397. doi: 10.1371/journal.pone.0247397 (PMC7924798; doi:10.1371/journal.pone.0247397)
Supplement: S2 File — (ZIP) [file pone.0247397.s002.zip › S1 File. Study aata/Health providers and Managers/Suggestions ways to improve reimbursement.docx]

[<Internals\\Health care providers\\IDI-Facility In-charge >](42a21635-e6b2-4991-a5d6-3deeba398cc3) - § 1 reference coded [5.57% Coverage]

Reference 1 - 5.57% Coverage

I What about your facility, is it NHIS accredited?

R We are working under the hospital and uses the hospital NHIS claims for our clients. So the CHPS must be made to be on its own and buy from medical stores directly and then submit claims for reimbursements and not going through the hospital, then the CHPS could get some funds to run the facilities well. I request that CHPS is accredited to NHIS and separately from the hospital.

[<Internals\\Health care providers\\IDI-Midwife-Deputy In charge ->](95069826-e33d-4365-91d6-3deeba72c532) - § 1 reference coded [12.44% Coverage]

Reference 1 - 12.44% Coverage

R The clients are not coming to the facility because of the environment. The weeds are taking over the place. The services are good but the environment deters people away. Also if there was to be a laboratory here, it will also help us a lot and we can save more clients. When clients come here and are told to go to Asuofia for laboratory, they usually don’t come back. They complain of the distance to travel and the facility is far from the community. Also the chieftancy is not helping us.

I What are they don’t doing to help you?

R Once you are in a community and they are not helping you, definitely you cannot survive. They don’t help with community engagements. I had worked in the Northern regions and the chiefs do organize community gathering and you go and give the community members heath talks but it doesn’t happen here.

I Don’t they organize durbar here?

R No, they don’t. I have been here for a year and not seen they organize any of such activities for us. They don’t come and talk to us and go and nothing happens. Rather people are building closer to the facility which is not advisable.

[<Internals\\Health care providers\\IDI-head of finance->](18b0e2f5-3dfc-4320-b9d6-3deebac1e6da) - § 1 reference coded [3.86% Coverage]

Reference 1 - 3.86% Coverage

Resp: With the reimbursement, the drug component and service component are paid together into one account and I think it not the best. If they pay everything into the service account there is a lot of pressure on the service account. So management may use some drug money for services activities whereby it affects the drug.

[<Internals\\Health care providers\\IDI-Hospital Accountant->](dfd23f0f-4df6-4615-82d6-3deebb1559a1) - § 1 reference coded [7.67% Coverage]

Reference 1 - 7.67% Coverage

R**:** yea recently they reviewed their medicine tariffs and instead of going upward they have rather come downwards. In Ghana we are not going downwards at all. Taxes are increasing everyday so bringing the medicine prices downwards they are compelling us to either we do top up payment, which they don’t want.

So we will now be sitting here prescribing for others to benefit. Which is bad. They don’t have prescribers there oo but we will sit here and prescribe for them then they will collect and put in their pocket. And we are sitting here which is very bad. And even causing inconvenience to the patient. If they think that they cannot review the price, they should open room for top up. I can’t go and buy medicine 10 cedis, health insurance is paying me 7 cedis. Then I buy the 10 and come and sit here and give it to the patients. Where are we going? Then the whole facility will come down so there is no sense in those things. If they think that they cannot increase it and the medicine is there then go to the market and come and give to the patient and we are given 7 cedis then the patient should pay the difference of the 3 cedi. And this is how they do it even that there is no magic inside because either there will be a damage, there will be a loss so even if you do that you have to add the margin so a at least it is 3 cedis. The patient will pay for that one and health insurance will pay for the other one. I think that will help. if they think they can’t review it upwards that is the only way to solve the problem.

[<Internals\\Health care providers\\IDI-Deputy Chief Health Service Adminstrator->](78faf6d3-b67b-41d9-8fd6-3deebb404497) - § 1 reference coded [8.97% Coverage]

Reference 1 - 8.97% Coverage

R What I will say is that insurance is now the life blood for health delivery, life blood for facilities and for the clients so the NHIA should be proactive in ensuring the sustainability of the health insurance. They should ensure frequent reimbursement and also review the tariffs upwards. At times over three years and the tariffs have not been reviewed and that affects service delivery. In Ghana every year there are increment in prices of goods and services and insurance will not review most of the tariffs and it becomes difficult to get the consumables for health care**.**

. For example if insurance is reimbursing me for 5 cedis when I bought it for 6 cedis and it becomes difficult. We request that the review should be done every year to commensurate the cost of living in the country. The central system of claims submissions delays so if they can equip the district to take back the mandate of claims management at the district level it will be better. Because we need to come to Kumasi and join queue whiles the district office is there. So NHIA should reconsider this issue again and come out with something substantial to minimize the challenges and burden on the only CPS here in Kumasi and it will be better. Thank you.

[<Internals\\Health care providers\\ IDI- Medical Superintendent - >](fe3c554a-3bb9-463f-a7d6-3deebb68cd65) - § 2 references coded [18.51% Coverage]

Reference 1 - 14.11% Coverage

R The NHIS should see us as partners in the care process. They at times see us as people who want to take their monies but that is not the case. But they have to see us as partners so we could work together. Though the law gives us the chance to follow up and ask if there has been deductions in your reimbursements, we don’t usually follow up on that. The other issue is that the payment should be regular so that we don’t run short of the commodities that we need for caring for patients. It will be sad that you go to a hospital and need care to survive and because they don’t have the resources to provide that service you will be at a disadvantage. Also

The NHIS should see us as partners in the care process and not see us as people who want to take their monies. Also, NHIS should look at the premium and let people pay realistic amounts so that they could get the needed services received. Also, the NHIA levy should be looked at and implemented effectively. The NHIS levy should be used for the needed purpose and not added to the consolidated funds that are used for other things. If the taxes collected for national health insurance are put in the NHIS fund and used correctly, they could be able to pay providers and care and services will go on smoothly. If the funds are divided into services and drugs at least we could get funds for each units and that will help the health insurance to move on perfectly.

Reference 2 - 4.39% Coverage

In addition, the premium charged for NHIS should be reviewed. There should be variations in the premium NHIS card holders pay because some people with chronic disease should be made to pay more since they do utilize health services the more and their costs are higher than others. People should be made to pay realistic prices and that will also help lessen the burden on health care providers. Thank you.

[<Internals\\Health care providers\\IDI-Deputy Chief Accountant->](51d2e908-a05b-49b2-81d6-3deebbec1627) - § 1 reference coded [10.69% Coverage]

Reference 1 - 10.69% Coverage

R NHIS has come to stay and I am of the view that the monies that NHIS gets is not enough to cater for the numerous clients who have been enrolled onto the system. So it is better for us Ghanaians to once again consider how best we can be able to support the NHIS to cater for everybody. Indeed you look at the fact that you go to the hospital and because you are card bearer, there is a service that cost like 10, 000 cedis but you are treated and you go free. So it is indeed a good social intervention but the funding that we are getting is not enough so all of us should come together to support the government to generate enough monies to continue to sustain the system. Again, once we are told somebody has been insured, I don’t see the need why we should demand co-payment because by so doing, we are not being fair to the clients. Though that could also bring us money, I see it not being fair to the clients and in order not to disobey the rules we should render efficient care to the clients. And once they come in their numbers, we will be able to get the necessary funding that we want to support our operations**.**

[<Internals\\Health care providers\\IDI- Deputy Chief Pharmacist- >](0274cbfb-ba52-4503-aed6-3deebc2ed937) - § 1 reference coded [4.35% Coverage]

Reference 1 - 4.35% Coverage

R What I want to add id that two captains cannot mann the same ship. Its highly impossible. The GHS won’t allow that they buy their drugs and then NHIS decide on how much to pay after giving to their clients. And the GHS is charging more than what NHIS will pay so because of that at the end of the day the clients are made to do co-payments which is not what we promised them. Sometimes the clients come and insult us that they are insured and we are taking their money to spend.

[<Internals\\Health care providers\\IDI- Health Service administrator->](bc053ab0-d4f7-4e92-9ed6-3deebc57616b) - § 1 reference coded [4.77% Coverage]

Reference 1 - 4.77% Coverage

R If the NHIS can speed up with payment and that will help a lot. I heard now they will make payment monthly and that will help us lot. Once they pay monthly, we will have funds available always to cater for emergencies. Also there are some projects that the NHIS is undertaking like renovation and outing up of new structures through the MPs common funds. There are some projects like in here the renovation of the maternity unit which started but not yet finished. We are left with the second phase so if NHIS can push in more money to get it completed and it will help a lot with care and service provision and more patient can benefit.

[<Internals\\Health care providers\\IDI- Medical Superintendent->](303021d4-6193-44b3-91d6-3deebc824cc9) - § 1 reference coded [11.68% Coverage]

Reference 1 - 11.68% Coverage

Thank you for your time. But any final words.

R For health insurance to go on, it needs to assess the health seeking behaviors of people. Health insurance practiced elsewhere cannot be brought here and imposed on us and no matter what you do, people will decide on where to get their services. The payment should be prompt and they should do their vetting well. They should verify claims and not put all facilities together and lament that they are not doing well. They have a monitoring unit but are not doing their work well of visiting the facilities to assess the folders but to say that some facilities are thieves is not the best. Now health insurance is beyond them and its in government control and I think the 2.5% of taxes should be enough to finance health insurance**.**

The challenge now is that the monies are put in the consolidated funds instead of a separate fund and invested. The NHIS funds are not invested and because it is in the consolidated funds, government can use it to pay salaries. If the health insurance money is to be used for only health insurance activities, we will not have any problem with reimbursement but used for other things. Now the whole process are centered in Accra and when the funds for NHIS from Ghana Revenue authority is released to the consolidated fund, its used for other things as well. Now we have to beg them to release money to us and because they are not releasing the money for us to work with, once we are not able to take good care of the clients, they are not able to say anything. Now we have to use co-payment and those who can afford to pay are given the services. Fortunately, people live to pay for their services and they are all alive in the system and telling them to pay for the services is not a difficult thing and that is what is sustaining the system and very soon they will die and go and the new ones will only know about health insurance. Thank you.

I End of Interview.

[<Internals\\Health care providers\\IDI- Maternity in charge->](1318a536-efbe-46e9-a3d6-3deebcaad598) - § 1 reference coded [10.05% Coverage]

Reference 1 - 10.05% Coverage

R The constant disbursement of funds is very necessary for the care and services given to our clients. Because when the disbursement is constant it helps us to develop a lot of things. For example this CCT machine, if the facility is well funded, we could get the CCT and can be co-payment and it will help. Because most people when you refer them on the account of the CCT they will ask you where they will be getting the money from for the service and I think it cost like 800 cedis. So if they don’t have the money, when they leave the facility, they don’t come back again. So these should be looked at. Thank you

[<Internals\\Health care providers\\IDI- Medical Sup In-charge->](0d23e9f1-c823-4c39-a6d6-3deebcd35d27) - § 1 reference coded [6.94% Coverage]

Reference 1 - 6.94% Coverage

R For the NHIS, we have a district office here and we fed up complaining to them that is why we are writing to the head office now. We have complained severally at the district office and nothing is coming out of it so we have stopped complaining there and now heading towards the head office in Accra. We hope this meeting will send information to the NHIS office to reimburse our monies. For care and service, we are treating patients and have not stopped. It’s the delay in reimbursing us with the money. When we were having the capitation, the monies didn’t delay at all until we got back to the NHIS and we are having delays in reimbursements and we don’t know the reasons why.

[<Internals\\Health care providers\\IDI- Deputy Director of Nursing Services->](44d0b877-60dc-446e-96d6-3deebd236d27) - § 1 reference coded [11.22% Coverage]

Reference 1 - 11.22% Coverage

R What i will say is that if we are doing health insurance, then we have to be up and doing because this is the health and lives of people so if facilities are not reimbursed it becomes difficult for them so the quality is a bit compromised and therefore government should try and reimburse them so they can do their work.

If they feel it cannot be done then they should find a way if people have to pay half and then they also pay the other half. Also those who can afford should be given the opportunity to do so and those who cannot afford, thus the poor will be given the NHIS. That is what I think and it’s a bit difficult because the preventive aspects don’t have the money to do their prevention. So doing education in other places become difficult and you can’t move and you are stuck at where you are. At times you use your own money to do what you want to do. So the government, other NGOs and partners should support the health sector to move forward. If we are able to prevent, we will not be in too much curative.
