# Supplementary material for: How does it affect service delivery under the National Health Insurance Scheme in Ghana? Health providers and insurance managers perspective on submission and reimbursement of claims
Source: PLoS One. 2021 Mar 2;16(3):e0247397. doi: 10.1371/journal.pone.0247397 (PMC7924798; doi:10.1371/journal.pone.0247397)
Supplement: S2 File — (ZIP) [file pone.0247397.s002.zip › S1 File. Study aata/NHIS Managers and claims officers/Challenges.docx]

[<Internals\\NHIS officers\\IDI-Claims officer-Regional Hospital->](92ece2c7-393b-40e2-a9d6-3deed7ac59ff) - § 4 references coded [15.53% Coverage]

Reference 1 - 3.91% Coverage

I Do you face challenges in the submission of claims.

R The challenge is that we don’t get the verification report and also with the e-claim people the policy is that when they are paying, they deduct 10% of the funds before payment is made. So that is the major challenge we have.

I Why do they deduct 10%?

R That is their policy and since they don’t have time to vet the submissions, they use their own judgement to pay the funds and deduct the 10% from it.

Reference 2 - 1.20% Coverage

I After the verification, will they pay back the 10% deductions?

R Yes they have to but we have not yet seen any payment of these deductions.

Reference 3 - 3.77% Coverage

I What are some of the background of the claim officers like you?

R Most of them are casual workers and others use the National service personnel to run the claims management. With that process it creates lots of problem because when the service personnel leaves, you have to train new sets of people to continue from the last point. So since NHIS has come to stay, we need to get staff to handle such roles whose work will solely be for claims.

Reference 4 - 6.66% Coverage

I So for your facility you don’t have issues with the signs and symptoms. Do you think these vetting team do reject your submissions because you had issues with signs and symptoms?

R Most of them are casual workers and others use the National service personnel to run the claims management. With that process it creates lots of problem because when the service personnel leaves, you have to train new sets of people to continue from the last point. Also the vetting team at the NHIS end they don’t have much clinicians there rather on the job training people. They are taught to match 1 to 2 so if its matched to 3, they complain its mis-match since they are not clinicians, it leads to the deductions. Also at the facility, the team working on claims submission makes mistakes due to the short hand writing by the clinicians. They write malaria as Mal or UTI and the new people will not know. Also the NHIS will not know the UTI as Urinary Tract infections. So these are some of the challenges that we face**.**

[<Internals\\NHIS officers\\IDI-NHIS Scheme Manager->](c4fa7ee5-476e-4108-a2d6-3deed83906b2) - § 2 references coded [8.35% Coverage]

Reference 1 - 3.17% Coverage

**Int:** What are the background of the claim of processes that you have at the health facility?

**Res:** The background actually some do not have the clinical eye. Some claim officers are doing quite well others too don’t have the clinical eye to be able to determine certain technical issues concerning health.

Reference 2 - 5.19% Coverage

**Res:** The claim submission is just sometimes as I said challenge is one the delay in submitting the claims that is one.

Secondly some providers also treat clients outside the medicine list they go contrary to the medicine list and we have a clinical audit team that frequently go around. When they come around they try to point out what some of these challenges to them and even sometimes those who affect the facility the facility will have to pay the money back to National Health Insurance Office.

[<Internals\\NHIS officers\\IDI- Hospital Claims officer->](022c13a8-1a9b-441f-add6-3deed85f2c12) - § 1 reference coded [6.37% Coverage]

Reference 1 - 6.37% Coverage

I Do you face challenges in the submission of claims.

R Sometimes it delays because we need to send some folders back to the NHIS for verification. At times the diagnosis and drugs given will not match so you need to send in the folder for verification. Also there might be occasions where the name, age and other items like the NHIS number not recorded for the client. These are information needed to process the claims. If these are not on the treatment card, you need to go for the folder for those information. At times too there are network problems that also delays us because we are sending the claims through the internet.

[<Internals\\NHIS officers\\IDI-NHIS Scheme Manager- >](1584bd65-84b1-4dad-99d6-3deed89ac86f) - § 1 reference coded [3.90% Coverage]

Reference 1 - 3.90% Coverage

First of all we cannot determine that they...the issue is that (interviewer cuts in with a probe: do they have a particular qualification to fit into …) that is not necessary. it’s about training. It is strictly by training definitely we expect that when it comes to some officers like medical officer you must have a medical officer. This one depending on the facilities you are running you are supposed to have x man or this type of medical officer. If you are running some equipment you must have the required number of professionals who are manning it. but when it comes to the processing of claims we need somebody who can really read and write and who understands the way the hospital is run and sometimes you can even ask somebody, another person who is free to come and help you. I know the field, we have not sanctioned it but I know people even on the field who go from hospital to hospital to do the processing of claims but that one part of the package we offer. Ours is to this is it. We teach you through it. But if you are not understanding we can still ask you to come back to us to retrain you but if you decide to go back to a calling then you should know that at the end of the month you are submitting it to our claim processing centre. We will do that actual thing. We will tell you this one is right, this one is not right and sometimes it is not always on the negative. Sometimes you can submit claims that show that you are even undercharged and we will adjust it well to fit what the correct tariff.
