# Supplementary material for: How does it affect service delivery under the National Health Insurance Scheme in Ghana? Health providers and insurance managers perspective on submission and reimbursement of claims
Source: PLoS One. 2021 Mar 2;16(3):e0247397. doi: 10.1371/journal.pone.0247397 (PMC7924798; doi:10.1371/journal.pone.0247397)
Supplement: S2 File — (ZIP) [file pone.0247397.s002.zip › S1 File. Study aata/NHIS Managers and claims officers/Guidelines.docx]

[<Internals\\NHIS officers\\IDI-Facility Claims officer->](92ece2c7-393b-40e2-a9d6-3deed7ac59ff) - § 1 reference coded [1.63% Coverage]

Reference 1 - 1.63% Coverage

I What are the guidelines for claims submission?

R We use the e-claims and we do that every three months through the NHIA CPC. We have a server and we submit through that and they rece7ive it.

[<Internals\\NHIS officers\\IDI-NHIS Scheme Manager->](c4fa7ee5-476e-4108-a2d6-3deed83906b2) - § 1 reference coded [4.71% Coverage]

Reference 1 - 4.71% Coverage

**I:** So, what are the guidelines guiding the submission of claims under the NHIS?

**R:** At least a claim should be submitted within three weeks. Every three weeks you should be able to prepare your claims and submit to the NHIA and roughly we also take some few days to process it and then maybe you are reimbursed. But some it takes more than three months before they submit claims to the National Health Insurance Authority and these causes’ delays.

[<Internals\\NHIS officers\\IDI-Facility Claims officer->](022c13a8-1a9b-441f-add6-3deed85f2c12) - § 1 reference coded [5.79% Coverage]

Reference 1 - 5.79% Coverage

I What are the guidelines for claims submission?

R When you finish with the claims, you add a cover letter and send the claims through internet to the claims office. Also, you have to send the soft copy on a CD rom to the claims office. There is a format for the cover letter and if you don’t follow that format, they will reject your submissions. There is a summary sheet that should be attached to the cover letter. This summary sheet explains all that the claims cover. Also, if there is no stamp on your letter and if there is no letter head too, they will not accept it.

[<Internals\\NHIS officers\\IDI-NHIS Manager->](1584bd65-84b1-4dad-99d6-3deed89ac86f) - § 1 reference coded [5.78% Coverage]

Reference 1 - 5.78% Coverage

What are the guidelines covering the submission of claims

I mean all facilities are… once they credential them there is a contract between us and the facilities. And once we credential you we have officers we have mounted supervision officers who will come and take you through the processes in terms of submission of claims. And then at the CPC we have the claims officers who will also take you through how you are supposed to prepare because the documents and everything all the credential things everything covering the way you can prepare, how to and so they tell you when you when you are offering services where to enter in your folder, how you are going to capture your prescription and how you are going to cover… and all these particulars are done. And at every office, every facility is supposed to have somebody in charge of claims because that is a specialized area it is not just a matter of who fills the forms. Because you must understand the knitty gritties of having to quantify both the services provided because health insurance is paying for both services and drugs. This is what we do. We are not just paying for medication. We are also paying for the services so you must have somebody who understands the tariffs that are charged on both what you call it that goes with the various ailments and how to calculate them. And once the person goes through that and is satisfied with what goes on he will sign and the medical director will also sign, counter sign and we require that you put it in a box, segregate it. Every box has what it takes month, what week by week. This week this is the number of people, and even within the box, the days Monday to Sunday so that when it comes it then becomes much easier for them to really do what take it and do the auditing and pass through the process. So when you do this and submit it then there are people who will be going to pick it and go through it. Then they are submitting so when you go through it (swallows words) so it is a huge office there are lots of people then they take you through all the processes before it goes through. So we teach them how to process their thing their claim so that at the end of the services they render we are going to pay for this particular service.
