# Supplementary material for: How does it affect service delivery under the National Health Insurance Scheme in Ghana? Health providers and insurance managers perspective on submission and reimbursement of claims
Source: PLoS One. 2021 Mar 2;16(3):e0247397. doi: 10.1371/journal.pone.0247397 (PMC7924798; doi:10.1371/journal.pone.0247397)
Supplement: S2 File — (ZIP) [file pone.0247397.s002.zip › S1 File. Study aata/NHIS Managers and claims officers/How often claims received.docx]

[<Internals\\NHIS officers\\IDI-Facility Claims officer->](92ece2c7-393b-40e2-a9d6-3deed7ac59ff) - § 1 reference coded [1.16% Coverage]

Reference 1 - 1.16% Coverage

I How often do you receive reimbursement for your claims?

R As at now they haven’t paid any 2018 submissions from January to July 2018 (IDI-39 yr old claims officer-AR).

[<Internals\\NHIS officers\\IDI-Scheme Manager->](c4fa7ee5-476e-4108-a2d6-3deed83906b2) - § 2 references coded [5.46% Coverage]

Reference 1 - 1.13% Coverage

**Int:** How often do you receive reimbursement for claims?

**Res:** Sometimes three months, sometimes four months

Reference 2 - 4.32% Coverage

**I:** So what is the most common one?

R: Between three to four months but the most common one is four months unless of course a particular facility has challenges with the claims.

like not doing the right thing, going outside the medicines list, not following what they are supposed to do and sometimes it delays authorities may have to detain the claims maybe come for clinical claims to be sure that the right thing is done before reimbursement.

[<Internals\\NHIS officers\\IDI-Facility Claims officer->](022c13a8-1a9b-441f-add6-3deed85f2c12) - § 1 reference coded [2.18% Coverage]

Reference 1 - 2.18% Coverage

I How often do you receive reimbursement for your claims?

R It takes two to three months before reimbursements are paid after submission. Since we received in November last year, we have not received any funds yet.

[<Internals\\NHIS officers\\IDI-NHIS Scheme Manager>](1584bd65-84b1-4dad-99d6-3deed89ac86f) - § 1 reference coded [1.37% Coverage]

Reference 1 - 1.37% Coverage

Sometimes depending on how quick you submit particulars sometimes you have a month. Of late we used to be challenged on money not being available, you know? We were told (fumbles with words) late last year that we were indebted if that is correct to the tune of almost 1.2 billion old Ghana cedis but (stammers) emm new Ghana cedis but we are almost the woods so it was difficult to fulfil some of our obligations towards . But I tell you since the middle of last year up to date some it is almost monthly. Sometimes twice in a month.
