# Supplementary material for: How does it affect service delivery under the National Health Insurance Scheme in Ghana? Health providers and insurance managers perspective on submission and reimbursement of claims
Source: PLoS One. 2021 Mar 2;16(3):e0247397. doi: 10.1371/journal.pone.0247397 (PMC7924798; doi:10.1371/journal.pone.0247397)
Supplement: S2 File — (ZIP) [file pone.0247397.s002.zip › S1 File. Study aata/NHIS Managers and claims officers/Reasons for delay reimbursement of claims.docx]

[<Internals\\NHIS officers\\IDI-Facility Claims officer->](92ece2c7-393b-40e2-a9d6-3deed7ac59ff) - § 2 references coded [6.00% Coverage]

Reference 1 - 2.03% Coverage

R There is delay alright from NHIS but the fault is from the providers. Most of them provide fake information to NHIS and that delays the payment. After submitting the claim the policy is that it takes three months before payments are made.

Reference 2 - 3.97% Coverage

I What are some of the reasons for the delay in reimbursements?

R Most of the institutions do not have the system to check the authentication of the ID so if the patient is holding the card on the system it will be inactive. So if it happens that way how can insurance get money to pay. No system for verification and its now that they are bringing verification machines. Once there is correct system to check ID authenticity then it could help reduce these challenges.

[<Internals\\NHIS officers\\IDI-NHIS Scheme Manager->](c4fa7ee5-476e-4108-a2d6-3deed83906b2) - § 2 references coded [14.38% Coverage]

Reference 1 - 8.56% Coverage

R: Actually, we know this affects the quality health care briefly, because you know sometimes, they have to stop their pharmacies to be able to provide essential drugs that they are all looking for. But sometimes the challenges also come the providers because the delay in submitting claims, sometimes they don’t submit claims at the right time they normally delay in submitting claims. So, once you delay, then our claims processing center CDC also delays. So when it happens that way before the head office get the information to process claims it takes time so sometimes the problem is from the providers and not from the national health insurance authorities even though we know there are challenges but mostly majority of the providers do not submit the claims at the right time. So that means it delays reimbursement.

Reference 2 - 5.82% Coverage

I: Are there issues that you have with your source of finance for reimbursing health providers

R: Generally, with NHIS when we started operations in 2004 we use to reimburse them timely depending on the timely support of claims. As the time goes on the membership keeps on increasing the utilization is going up and for that matter there is that kind of funding gap and ones there is a funding gap it means that something must be done about it to ensure prompt payment of claims. They must get additional source of funding to be able to sustain the NHIS.

[<Internals\\NHIS officers\\IDI- Facility Claims officer->](022c13a8-1a9b-441f-add6-3deed85f2c12) - § 2 references coded [8.08% Coverage]

Reference 1 - 3.84% Coverage

I What are some of the reasons for the delay in reimbursements?

R There are lots of facilities submitting to the CPC and they also have to process it. They vet, write report and then send to Accra to reply. If there are mistakes in the report, they have to do it again and submit. Also the CPC office is only one and Brong Ahafo region facilities also submit to Kumasi CPC office.

Reference 2 - 4.24% Coverage

I Do you think that knowledge for the signs and symptoms have also led to the claims being rejected?

R That one too is true because if you don’t have any idea on those things, you will just do a rough thing but if they had knowledge on the signs and symptoms then they will know this is right and that is wrong. So we are trying to get a fair knowledge on science and technology to help the system address those issues.

[<Internals\\NHIS officers\\IDI-NHIS manager->](1584bd65-84b1-4dad-99d6-3deed89ac86f) - § 2 references coded [3.31% Coverage]

Reference 1 - 1.12% Coverage

You have to provide the service and once you have provided the services, you bring the claims because we are not the ones to chase you for them. So once it comes to use we have to pay but most of the problems we have had in the Northern region here had to do with the delay in submission claims. Secondly, improper processing of claims by the health facilities delays the process.

Reference 2 - 2.18% Coverage

The delays we said that if you don’t submit early we cannot be able to pay. The thing, everything is based on the claims that has been what processed. Earlier submitted, processed without problems because once we are processing them and there is a problem, we will call you to come and pick your package and go and to the right things. So, submission of early claims. Really following the regulation that has been set in terms of how to present your claims. Once you do it early and it comes early there are no problems, be sure that you are going to get it. but if there are a lot of problems which we normally encounter definitely you may have submitted it very early but if it doesn’t meet the dictates definitely you will have to come and sometimes people come 2, 3 times just to correct one single mistake that has been explained over and over again.

[<Internals\\NHIS officers\\IDI-Facility Claims Officer->](87e530d2-6206-4612-93d6-3deed8c5b22a) - § 3 references coded [19.68% Coverage]

Reference 1 - 8.31% Coverage

I So do you think that the knowledge of the signs and symptoms of disease have accounted for the claims being rejected and also some of the challenges you face in the submission of claims.

R Yes it could be. Some of the report shows unclear diagnosis like you write trauma, what is it? As a national service person, I just key in trauma or skin infection which are not technical term but something they keep writing. Now NHIS will be there and wonder what kind of skin infections are that. If it is trauma like road traffic accident (RTA), you indicate it, domestic assault but as the doctor has written it then they key it in. But if its someone who has been in the system for long will know that those terms are used but not acceptable.

Reference 2 - 6.71% Coverage

I What are some of the reasons for delay in reimbursement for service provided?

R I think some of the issues come from NHIS. About 90% of Ashanti, Brong-Ahafo and West Regions submitted their claims as though the claims processing centre which is wrong. Having three big regions submit at the same is not the best. It will go through vetting and I like the control measures but it will take over three months to be processed and we too might have used three months to prepare so that mean six month of delay funds. They should work on the vetting process and at least it should be regional based.

Reference 3 - 4.66% Coverage

I Do you also think it is as a result of inadequate financing?

R That is the backbone of all the challenges and I have an issue like you need to ignore all these private clinics and hospital aside. It is in adequate of finance so you could have pre-financed so that after vetting and it is over paid, the subsequent months, it will go against the hospital. Since there is no funds for that, lets us such strategy.
