# Supplementary material for: How does it affect service delivery under the National Health Insurance Scheme in Ghana? Health providers and insurance managers perspective on submission and reimbursement of claims
Source: PLoS One. 2021 Mar 2;16(3):e0247397. doi: 10.1371/journal.pone.0247397 (PMC7924798; doi:10.1371/journal.pone.0247397)
Supplement: S2 File — (ZIP) [file pone.0247397.s002.zip › S1 File. Study aata/NHIS Managers and claims officers/Suggested ways to improve NHIS and reimbursment of claims.docx]

[<Internals\\NHIS officers\\IDI-39 yr old Claims officer-Regional Hospital-AR>](92ece2c7-393b-40e2-a9d6-3deed7ac59ff) - § 1 reference coded [7.88% Coverage]

Reference 1 - 7.88% Coverage

R NHIS do not have a system to tract and check their clients and need to get one like what the banks are doing and you can monitor transactions. It is only when claims are submitted that NHIS knows a client has utilized services. We need one platform where if a client visits a facility and keys in, it shows that such services have been provided to the client. There is nothing like that in the system to track transactions. A client might visit several facility for the same treatment and at the end NHIA loses but the facilities gains. This if not well coordinated could collapse NHIA**.**

But NHIS is good and if you don’t have insurance and you are admitted here at this facility for a week you will pay like 1000 cedis and those insured will be only 102 cedis. So the NHIS need one big platform to coordinate activities else the system if not well managed will collapse.

[<Internals\\NHIS officers\\IDI-NHIS Scheme Manager->](c4fa7ee5-476e-4108-a2d6-3deed83906b2) - § 1 reference coded [5.50% Coverage]

Reference 1 - 5.50% Coverage

**Res:** Actually the sustainability of the scheme depends on all of us and the major stakeholders. It does not depend only on the health insurance management it depends on all the major stakeholders especially all the health providers. They have a special role to play to ensure the sustainability of the scheme because they are providing the services and they have to ensure that the right thing is done

then effectively collaboration is done with the other stakeholders then the sustainability of the national health is reached.

[<Internals\\NHIS officers\\IDI- Facility Claims officer->](022c13a8-1a9b-441f-add6-3deed85f2c12) - § 1 reference coded [6.70% Coverage]

Reference 1 - 6.70% Coverage

R I want to talk about the service tariff and this what we have been using since 2016. If they can review the tariff for us, it will help a lot. Also if the reimbursement could come frequently, then it will help a lot and there will not be co-payments**.**

Also if there could be CPC (claims processing centre) at every center, it will really help because traveling all the way to Kumasi from Sunyani to submit claims is risk, use of resources and other costs. But if office is in Sunyani it will help to speed up the processes. Also if they are able to establish offices in those regions, it will help address lots of problems we face here in the imbursement process.

[<Internals\\NHIS officers\\IDI-NHIS Manager>](1584bd65-84b1-4dad-99d6-3deed89ac86f) - § 1 reference coded [2.66% Coverage]

Reference 1 - 2.66% Coverage

What I will only ask is … I will add is just that all Ghanaians must support this thing to make it very sustainable. Like I said, this health insurance I am yet to read across the world of any health insurance package that provides more than the Ghanaian model. Ours was considered because we had to combine different models of health insurance and arrive at this particular model. That will then serve our people well because so we must support it. We must make sure that we keep it so that it becomes more sustainable in terms of our finances because once we have more finances, funding coming from all our fund (swallows’ word) sources whether it is SSNIT, from the general populace I think we can offer better services. Sometimes the issue about not being able to offer the best of care is just because we do not have funding. So when the need arises we will urge all Ghanaians to support us and to support us through their contributions to make sure that we make health insurance last forever and care for future generations.
