# Supplementary material for: How does it affect service delivery under the National Health Insurance Scheme in Ghana? Health providers and insurance managers perspective on submission and reimbursement of claims
Source: PLoS One. 2021 Mar 2;16(3):e0247397. doi: 10.1371/journal.pone.0247397 (PMC7924798; doi:10.1371/journal.pone.0247397)
Supplement: S3 File — (DOCX) [file pone.0247397.s003.docx]

**Interview Guide for Health Care Providers/Managers**

The School of Public Health of University of Ghana wishes to interact with you to understand out of pockets payments occur in your facility.

**Background data of respondent**

Type of health facility:

Years of service at facility:

Age:

Sex:

Rank:

NHIS Accreditation year of Facility:

Current status with NHIS:

1. **Health care services**
   1. What services do you provide at your health facility?
   2. Which services are patronized more? Why?
2. **Clients of NHIS**
   1. Among the clients accessing health care at this health facility what is your perception of NHIS clients receiving care here?
      1. What is their pattern of utilization of care?
      2. What services do they patronize more?
      3. At what stage of illness do most come to the facility for care?
      4. What common illnesses do they present with?
      5. What is their attitude towards you care you provide them?
3. **Services charged**
   1. What services do you charge your clients for?
   2. Is it the price per service charged the same for both insured and uninsured clients?

Probe: If No, how much more do the uninsured pay?

How much more do the insured clients pay?

What services do you ask those insured to pay out of pocket for?

How often do they pay these services out of pocket?

What are the reasons why you sometimes charge those insured to pay out-of-pocket?

What co-payment system does you facility have?

What necessitated the copayment system in your facility?

1. **Claims submission and reimbursement:**

- What are the guidelines governing submission of claims?
- What has been some of the challenges in claims submission in this facility?
- How often do you receive reimbursement for claims submitted?
- What are your experiences in receiving reimbursement for the claims you submit to NHIS?
- Probe: If delay for reimbursement is mentioned, find out when was the last time facility was reimbursed
- How have these experiences affected services you provide in this facility?
- How have you treated insured patients due to your experiences with reimbursement of claims?
- Generally, what do you think could be done to improve on reimbursement of claims and service delivery under the NHIS?

**Thank you very much for participating in this study**
