# Supplementary material for: How does it affect service delivery under the National Health Insurance Scheme in Ghana? Health providers and insurance managers perspective on submission and reimbursement of claims
Source: PLoS One. 2021 Mar 2;16(3):e0247397. doi: 10.1371/journal.pone.0247397 (PMC7924798; doi:10.1371/journal.pone.0247397)
Supplement: S4 File — (DOCX) [file pone.0247397.s004.docx]

**Interview Guide for NHIS Managers**

The School of Public Health of University of Ghana wishes to interact with you to understand better how out-pocket of payments by insured clients occur in the facilities

**Background data of respondent**

District:

Years of service as district Scheme Officer:

Age:

Sex:

Rank:

**a. Resources for NHIS service**

1.There have been media reports and published information that insured clients of the NHIS are treated unfairly when they seek care at credentialed facilities. What are your opinions on why they are treated unfairly?

1. In what ways are they treated unfairly?

Probe: if delays in reimbursement are mentioned asked how long have there been delays in reimbursement to providers? Which year did the late reimbursement start?

What are the reasons for the delays in reimbursing providers for services they provide?

Probe: If inadequate finance is mentioned, ask what are the sources of funds for the NHIS?

What are the targets set for each financing source? Are the targets exceeded? If Yes/No, explain

What are the drivers for the costs of care for services provided by credentialed health facilities?

There anecdotal evidence that clients of NHIS pay services out of pockets? Why do you think this is so?

What services do they pay out of pocket for? What reports have clients laid with respect to paying out of pocket officially?

In your opinion, the tariffs set for each service, how does it affect the market price for services providers provide?

What do you think are the actual costs of providing care under the NHIS?

How does your institution propose to remove out-of-pocket payments by insured clients?

Generally, what do you think could be done to improve on reimbursement of claims and service delivery under the NHIS?

**Thank you very much for participating in this study.**
